# Macrophage-derived exosomes in bone tissue regeneration: spatiotemporal immunomodulation, engineering advances, and translational frontiers

**DOI:** 10.1186/s12967-026-08613-0

**Published:** 2026-07-25

**Authors:** Shuang Lai, Xinxiong Xia, Yandong Mu

**Affiliations:** Department of Stomatology, Sichuan Provincial People’s Hospital, School of Medicine, University of Electronic Science and Technology of China, Chengdu, 610054 China

**Keywords:** Exosomes, Macrophages, Bone regeneration, Immunomodulation, Tissue engineering

## Abstract

**Background:**

Bone regeneration unfolds through sequential inflammatory, reparative, and remodeling phases, with macrophages functioning as pivotal regulators across these stages. Accumulating evidence identifies macrophage-derived exosomes (M-Exos) as key mediators of intercellular communication that dynamically shape the bone healing microenvironment.

**Main body:**

This review synthesizes current evidence on the spatiotemporal roles of M-Exos during bone healing and discusses how donor macrophage states may shape vesicle function across inflammatory, reparative, and remodeling phases. The discussion elucidates how dynamic macrophage functional states shape exosomal cargo and biological activity during distinct stages of repair. Concurrently, emerging engineering strategies to harness and enhance M-Exos’ regenerative functions are evaluated, including exosome engineering, therapeutic cargo optimization, and biomaterial-assisted delivery approaches. In addition, this paper scrutinizes the translational landscape of M-Exos, emphasizing their potential as a cell-free therapeutic modality for bone tissue engineering while outlining key challenges critical for clinical implementation.

**Conclusions:**

By integrating the spatiotemporal functions of macrophage-derived exosomes (M-Exos) and single-cell-resolved landscapes of macrophage functional states, this review delineates the mechanistic links among macrophage state transitions, M-Exo cargo profiles, and regenerative outcomes across distinct phases of bone healing. It further provides a theoretical basis for developing stage-adaptive and functionally tunable M-Exo–based strategies for bone regeneration.

**Supplementary Information:**

The online version contains supplementary material available at https://doi.org/10.1186/s12967-026-08613-0.

## Background

Although bone possesses an intrinsic capacity for regeneration, complex fractures and critical-sized defects frequently override these self-repair mechanisms [1], resulting in impaired healing or nonunion in 5–10% of cases globally [2]. The bone-healing cascade proceeds through overlapping inflammatory, soft-callus, hard-callus, and remodeling phases, and its successful progression depends on tightly coordinated spatiotemporal regulation of the osteoimmune microenvironment [3]. Upon skeletal injury, the immediate recruitment and activation of immune cells initiate the repair cascade [4], among which macrophages have emerged as central orchestrators whose continuous phenotypic plasticity governs the transition from initial inflammation to definitive tissue regeneration [5].

Accumulating evidence identifies macrophage-derived exosomes (M-Exos) as vital vesicular conduits mediating multicellular crosstalk within this bone-repair niche [6]. By transferring bioactive proteins, lipids, and regulatory RNAs [7], M-Exos enable macrophages to communicate with mesenchymal progenitors, endothelial cells, osteoblast-lineage cells, and osteoclasts, effectively coupling immune regulation with skeletal remodeling [8]. Crucially, high-resolution single-cell and spatial transcriptomic atlases have dismantled the classic binary M1/M2 polarization paradigm, revealing a spectrum of specialized macrophage states—including inflammatory, interferon-responsive, phagocytic, angiogenic, matrix-remodeling, and pro-resolving populations—that dynamically evolve across the bone-repair timeline [9]. Consequently, M-Exos must be conceptualized as state-dependent, highly dynamic vesicular outputs whose cargo profiles and functional repertoires mirror the shifting polarization programs of their donor cells.

Despite these insights, significant knowledge gaps persist regarding the precise mechanisms through which distinct macrophage state programs dictate exosomal cargo sorting, and how these vesicles achieve stage-specific coordination of immune resolution, angiogenesis, and osteogenesis in vivo. Current literature relies heavily on static, discrete-time-point evaluations or simplified in vitro polarization models, which fail to capture the fluid osteoimmune dynamics that determine clinical outcomes. This mechanistic obscurity remains a major bottleneck for the rational bioengineering of M-Exos-based therapeutics, particularly for strategies that require phase-matched immunomodulatory delivery rather than non-specific vesicle administration.

This review comprehensively synthesizes the spatiotemporal immunomodulatory functions of M-Exos throughout the bone-regeneration cascade. By integrating classical bone biology with recent single-cell and spatial transcriptomic datasets, we delineate how donor-macrophage states translate into therapeutic cargo profiles that regulate niche-specific cellular crosstalk. Furthermore, we evaluate advanced engineering strategies—including donor-cell preconditioning, cargo enrichment, and biomaterial-driven delivery—designed to enhance the precision and potency of M-Exos. Finally, we address the critical translational bottlenecks and scalable manufacturing challenges that must be resolved to transition M-Exos into predictable, cell-free immunomodulatory therapies for bone repair.

## Biological landscapes and state-resolved functional basis of M-Exos

### Definition, biogenesis, and characteristics of exosomes

Exosomes are ~50–150 nm endosomal extracellular vesicles released through multivesicular bodies [10] and commonly identified by canonical markers such as CD9, CD63, CD81, ALIX, TSG101 [11, 12]. Macrophages actively release exosomes during inflammation and tissue repair, using these lipid-bilayer vesicles to transfer regulatory RNAs, proteins, cytokines, enzymes, and lipids to immune and stromal target cells [13, 14]. For M-Exos, the key biological issue is therefore not only vesicle identity [15], but how the state, ontogeny, and niche exposure of the parent macrophage shape cargo selection and downstream immunomodulatory activity [16].

### Surface markers and molecular cargo of M-Exos

**Surface Markers**: M-Exos display characteristic surface markers common to exosomes as well as proteins reflective of their cellular origin [17]. General exosome markers include the tetraspanins CD9, CD63, and CD81, and membrane-associated proteins like ALIX and TSG101 [7]. These are routinely used to identify and isolate exosomes. In addition, exosomes from macrophages can carry immune-related surface molecules [18]. For example, major histocompatibility complex (MHC) class II molecules have been found on exosomes released by dendritic cells and macrophages, enabling these vesicles to participate in antigen presentation and immune signaling [19]. M-Exos may also display polarization markers or receptors from their cell of origin. These proteins can be incorporated into released exosomes, potentially influencing which cells or tissues the exosomes interact with. For instance, ICAM-1 and integrins on the surface of M-Exos mediate selective adhesion to recipient cells. Exosomal α4β1 integrin may favor interactions with vascular cell adhesion molecules in injured bone marrow, whereas ICAM-1 facilitates endothelial engagement [20].

**Molecular Cargo:** Internally, M-Exos are loaded with diverse molecular cargo, including proteins, nucleic acids, and lipids that mirror the functional state of the parent macrophages. Key cargo components include:

**Regulatory RNAs:** M-Exos are particularly enriched in microRNAs (miRNAs), small non-coding RNAs (~22 nt) that post-transcriptionally regulate gene expression [21]. They often carry miRNAs that modulate inflammatory and regenerative pathways. For example, M2-Exos tend to contain anti-inflammatory or pro-resolving miRNAs such as miR-21b [22], miR-146a [23], and miR-223 [24], which can suppress pro-inflammatory signaling in target cells and promote tissue repair. In contrast, M1-Exos are enriched in miRNAs like miR-155 [25] and miR-125a-5p [26] that promote inflammation and can inhibit regenerative processes. These miRNAs, once delivered to recipient cells, alter the expression of genes involved in inflammation, apoptosis, and differentiation. Exosomes also carry other non-coding RNAs such as long non-coding RNAs, circular RNAs, and messenger RNAs (mRNAs) [27]. Notably, mRNAs packaged in M-Exos can be translated in recipient cells, yielding functional proteins. An example is IL-10 mRNA found in M2-Exos, which can be delivered to other cells and translated, thereby inducing production of the anti-inflammatory cytokine IL-10 in the target cell [28].

**Proteins and Enzymes:** M-Exos carry a wide array of proteins, including cytokines [29], growth factors, enzymes, and signal transducers. While only a small fraction of any given cytokine may be exported via exosomes, the vesicles have been shown to contain proteins such as TNF-α, IL-10, and TGF-β1, reflecting the macrophage’s polarization state. M1-Exos can carry pro-inflammatory cytokines or components of inflammatory signaling complexes, whereas M2-Exos are more likely to contain anti-inflammatory mediators and tissue repair enzymes. For instance, macrophage exosomal proteins include enzymes that remodel the extracellular matrix or influence signaling pathways such as kinases and ubiquitin-modifying enzymes. A striking example is the deubiquitinase CYLD, identified in exosomes from M2 macrophages, which can attenuate NF-κB signaling in osteoclasts to inhibit bone resorption [30].

**Lipids:** Exosomal membranes are notably enriched in particular lipids such as cholesterol, ceramides, sphingomyelin, and other sphingolipids. These lipids are not mere structural components; they can modulate signaling and membrane fusion in target cells [31]. Sphingolipids and ceramides carried by exosomes, for example, may influence cell death pathways or serve as bioactive mediators when delivered to recipient cells [32]. Additionally, the lipid composition of exosomes affects their uptake; high cholesterol content can promote fusion with target cell membranes, and certain lipids can target exosomes to lipid raft microdomains on the recipient cell [33].

### Polarization-dependent cargo profiles and functional heterogeneity of M-Exos

**Fig. 1:**
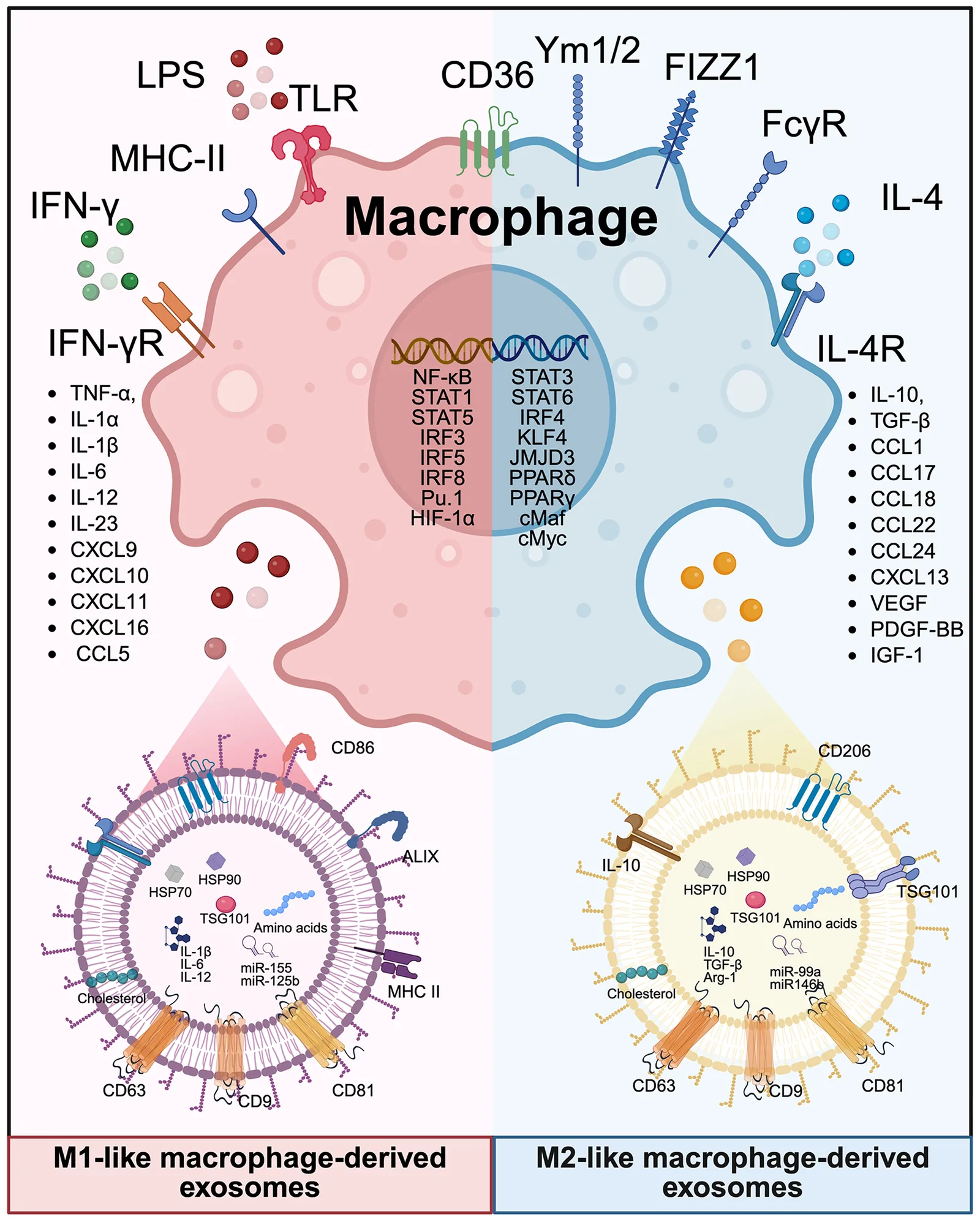
Distinct molecular signatures of exosomes derived from M1 and M2 polarized macrophages. Schematic illustration depicting the unique surface markers and bioactive cargo of exosomes originating from classically activated (M1) and alternatively activated (M2) macrophages. M1-derived exosomes (M1-exos) are enriched in pro-inflammatory surface molecules (e.g., CD86, MHC II) and internal cargo including cytokines (TNF-α, IL-6) and microRNAs (e.g., miR-155, miR-222), which amplify inflammatory signaling and suppress osteogenic processes. Conversely, M2-derived exosomes (M2-exos) harbor anti-inflammatory and pro-regenerative components, such as cytokines (IL-10, TGF-β1), growth factors (e.g., VEGF), and microRNAs (e.g., miR-21, miR-146a, miR-223), facilitating immunomodulation, tissue remodeling, and bone formation. Both subtypes express common exosomal markers (e.g., CD63, CD81, TSG101), underscoring their shared biogenesis while highlighting polarization-dependent functional divergence for targeted therapeutic exploitation in bone regeneration

Macrophage polarization has a profound effect on exosome content and function. Classically activated M1 macrophages and alternatively activated M2 macrophages remain useful experimental anchors, but recent single-cell studies indicate that macrophage identity is better understood as a continuum of developmental, inflammatory, pro-resolving, angiogenic and matrix-remodeling states rather than a strict binary [34]. This state-continuum concept is directly relevant to M-Exos because transcriptional, metabolic and tissue-niche programs in donor macrophages influence vesicular miRNA, mRNA, protein and surface-marker cargo [7]. In other words, single-cell atlases define the donor-cell states, EV biology explains how these states can be encoded into vesicles, and M-Exos experiments test whether the transferred cargo reprograms recipient cells. Broadly, M1-Exos tend to carry cargo that propagates inflammation or impedes tissue regeneration, whereas M2-Exos carry signals that resolve inflammation and promote repair, as shown in Fig. 1. Accordingly, M-Exos should be interpreted as state-encoded vesicular outputs, in which donor-cell transcriptional programs shape vesicle biogenesis and cargo selection, and the resulting RNA/protein payload reprograms recipient macrophages, mesenchymal progenitors, endothelial cells, and osteoclast precursors in a phase-dependent manner. Single-cell macrophage atlases can therefore serve as practical roadmaps for M-Exos engineering by identifying donor states to enrich, inflammatory cargo modules to avoid, and pro-resolving or pro-regenerative cargo modules suitable for therapeutic delivery.

**Pro-inflammatory M1-Exos:** Under inflammatory stimuli, M1 macrophages package a pro-inflammatory repertoire into their exosomes. These M1-Exos are enriched in miRNAs and cytokines that can exacerbate inflammatory responses or induce apoptosis in certain targets. For example, M1-Exos produced under hypoxic or stress conditions have high levels of miR-222, a microRNA known to trigger apoptosis in mesenchymal stem cells (MSCs) by suppressing the anti-apoptotic protein Bcl-2 [35]. Similarly, M1-Exos are enriched in miR-155, a prototypical pro-inflammatory miRNA. Exosomal miR-155 delivered to osteogenic cells has been shown to downregulate key transcription factors for osteoblast differentiation and to promote osteoclastogenesis, skewing the balance toward bone resorption. In a bone injury context, M1-Exos containing miR-155 were found to inhibit bone morphogenetic protein (BMP) signaling in progenitor cells, thereby reducing new bone formation [25]. Beyond miRNAs, M1-Exos carry inflammatory cytokines (like TNF-α and IL-6) and can present surface molecules (e.g., CD86, MHC II) that stimulate immune cells. The net effect of M1-Exos on target cells often includes increased expression of inflammatory genes, induction of apoptosis or senescence in regenerative cells, and interference with pro-healing pathways. These findings align with the concept that M1-Exos act as “injury-spreading” vectors in chronic inflammation or tissue damage settings.

**Anti-inflammatory M2-Exos:** In contrast, M2-polarized macrophages secrete exosomes loaded with anti-inflammatory and pro-regenerative signals. M2-Exos are rich in miRNAs that dampen inflammation (e.g., the let-7 family [36], miR-21 [37], miR-146a [23], miR-26 [38]) and in mRNAs/proteins that support tissue remodeling and growth. For instance, M2 exosomal miR-223 can target the NF-κB pathway in recipient inflammatory cells, thereby reducing pro-inflammatory cytokine production and promoting a healing phenotype [24]. Notably, M2-Exos carry the anti-inflammatory cytokine IL-10, which they can deliver to target cells to boost local IL-10 levels and resolve inflammation [39]. Additionally, growth factors such as TGF-β1 and VEGF can be packaged into M2-Exos, contributing to angiogenesis and matrix deposition in healing tissues [40]. Functionally, M2-Exos have demonstrated direct pro-regenerative effects in bone tissue engineering models by promoting osteoblast activity while concurrently inhibiting osteoclast development. Kang et al. showed that macrophage extracellular vesicles exert polarization-dependent effects in bone regeneration: M1-like vesicles enriched in inflammatory cargo can suppress osteogenic signaling, whereas M2-like vesicles enriched in miR-378a activate BMP signaling in MSCs and enhance osteogenic differentiation [41]. M2-Exos not only act on tissue progenitors but can also re-educate other immune cells; in diabetic fracture healing, M2-Exos reduced M1 macrophage proportions, increased M2 macrophage abundance and improved callus repair through PI3K/Akt-associated immunomodulation [42].

**Functional Heterogeneity and Single-Cell Guidance:** Even exosomes derived from macrophages assigned to the same polarization category are not homogeneous [43]. Heterogeneity in vesicle size, composition and bioactivity arises from the subcellular origin of vesicles, the stimuli encountered by donor macrophages, and donor-to-donor differences [33]. Tissue macrophage biology is highly niche-dependent, and recent reviews emphasize that local signals can imprint specialized macrophage programs that cannot be fully captured by the M1/M2 framework. Single-cell and proteomic analyses have therefore become important for linking macrophage states to EV function [44, 45]. For example, Dechantsreiter et al. used super-resolution microscopy and mass spectrometry to show that M0-, M1- and M2-like human macrophages secrete EVs with distinct size distributions and protein compositions. Their proteomic profiling showed that M1-like EVs are enriched in pro-inflammatory proteins such as IL-18, pro-IL-16 and HLA-DR, whereas M2-like EVs carry anti-inflammatory and tissue-reparative markers including CD163, CD180, SMAD2 and folate receptor beta [6]. Thus, high-resolution macrophage atlases can help predict which exosomal cargo modules should be enriched or excluded when designing immunomodulatory M-Exos. Single-cell atlases alone do not prove the therapeutic efficacy of a given M-Exos product; rather, they identify candidate donor states and marker programs that must be paired with EV cargo profiling and functional assays. For example, inflammatory-risk modules such as S100A8/S100A9/VCAN/CXCL8/HLA-associated programs [46], tolerogenic or pro-resolving modules such as MARCO/CD5L/VSIG4/HMOX1/MRC1/CD163 [47, 48], and immunoregulatory surface modules such as CD206/FOLR2/LYVE1 [49, 50] can be translated into donor-cell qualification panels. State-resolved macrophage atlases can help nominate donor-cell states, inflammatory-risk modules, and candidate signaling programs for M-Exos engineering.

Taken together, these state-resolved datasets provide the conceptual bridge for the immunomodulatory discussion below: they should be used as hypothesis-generating maps rather than as direct proof of exosomal cargo. Such datasets nominate macrophage donor states, ligand-receptor axes, and candidate cargo modules; exosomal RNA/protein profiling and functional potency assays are then required to confirm whether the resulting M-Exos actually reprogram recipient macrophages and support phase-matched bone repair.

### Advanced isolation and characterization techniques of M-Exos

For both mechanistic and therapeutic development, the isolation and characterization of M-Exos must be sufficiently rigorous to preserve vesicle integrity while accounting for macrophage-state-dependent heterogeneity. Different macrophage states may release vesicle populations with distinct cargo profiles and biological activities; therefore, M-Exos preparation should not only confirm generic exosome identity but also consider whether the isolated vesicles retain the intended macrophage-derived and state-associated features [47]. Table 1 summarizes commonly used isolation strategies and key considerations for M-Exos preparations.

**Table 1:** M-Exos separation and enrichment strategies with translational and clinical considerations

Isolation Strategy	Principle	Main advantages	Key limitations	Clinical Suitability	Considerations for M-Exos	Reference
Differential Ultracentrifugation	Sequential high-speed centrifugation pelleting vesicles according to size and density, often above 100,000 ×g.	Historically established and widely used; can process moderate to large culture volumes; dose not require complex exogenous reagents.	Labor-intensive and low throughput; co-pellets protein aggregates, debris, and lipoproteins; high shear can damage membranes.	Low; difficult to scale under GMP-compatible sterile manufacturing and prone to batch variability.	Requires EV-depleted serum or serum-free workflows, otherwise exogenous vesicles can contaminate the macrophage-derived transcriptomic background.	[48–50]
Density-Gradient Ultracentrifugation	Sucrose or iodixanol gradients separate vesicles by buoyant density, typically around 1.08–1.18 g/ml.	Yields highly pure exosome fractions (separates vesicles from most impurities); avoids some vesicle damage by flotation method.	Extremely time- and labor-intensive (>16 h centrifugation); low recovery yield (multi-step process); requires ultracentrifuge and careful gradient preparation.	Very limited – Impractical at large scale (slow and low-yield); mainly a research tool for purity.	Critical for state-specific proteomics or small-RNA sequencing because it reduces background from soluble inflammatory mediators.	[12, 48, 51]
Size-Exclusion Chromatography (SEC)	Size-based separation through porous polymer resin: exosome-sized particles elute first, smaller proteins elute later	Gentle, non-destructive to exosomes (preserves integrity and bioactivity); effectively removes most soluble protein contaminants (e.g. albumin, free cytokines); simple and reproducible.	Dilutes the exosome sample (eluted in volumes); limited loading capacity per column – large volumes require multiple runs; an extra concentration step is often needed after collection.	Moderate – already used in semi-automated setups; feasible for clinical prep as a polishing step (uses no harsh chemicals), but scaling to very large volumes may require parallel columns.	Especially useful for removing macrophage-secreted soluble cytokines such as TNF-α and IL-1β that could confound immunomodulatory assays.	[52]
Tangential Flow (TFF)	Flow parallel to the membrane concentrates vesicles while removing solvent and small molecules through ultrafiltration membranes, commonly around 100 kDa cutoff.	Fast and scalable concentration of large volumes; high recovery yield (minimal vesicle loss); closed-system processing available (GMP-compatible).	Non-specific – concentrates all particles above the pore size (can retain protein complexes, lipoproteins); membrane clogging possible (mitigated by tangential flow).	High – Well-suited for clinical manufacturing (already used to concentrate EV batches); often integrated in production workflows (followed by SEC or other purification).	Well suited for primary concentration and buffer exchange of macrophage-conditioned media from bioreactors or hollow-fiber systems.	[47]
Polymer Precipitation (PEG)	Addition of volume-excluding polymers (e.g., polyethylene glycol) that reduce exosome solubility, causing vesicles to pegylate and precipitate for collection by low-speed centrifugation.	Very simple protocol (mix-and-incubate); no expensive equipment needed; can handle large sample volumes in parallel; high exosome yield (efficient recovery)	Low purity – co-precipitates many non-EV components (proteins, polymer carryover); precipitant must be removed from final prep; variability in precipitating conditions can affect reproducibility	Low – Not ideal for clinical-grade isolation due to residual polymer and contaminants; typically used as a preliminary enrichment in research.	Macrophage secretomes are rich in soluble proteins, making PEG precipitation especially problematic for BMSC osteogenesis or immunomodulation assays.	[53]
Immunoaffinity Capture	Antibody-based capture of exosomes via surface markers: antibodies against exosomal proteins (e.g. CD9, CD63, CD81 or cell-specific markers) immobilized on beads or columns selectively bind target exosomes	Extremely high purity isolation (near-homogeneous exosome population); enables enrichment of specific exosome subtypes (e.g. use anti-CD11b or anti-MHC-II to capture only M-Exos)	Not all exosomes display the target marker – a subset may be missed; this method typically results in a low yield; requires large starting volumes; antibodies and matrices are costly; not easily scalable for bulk production	Low – Primarily for analytical/diagnostic prep where purity or specific subpopulation is paramount; impractical for manufacturing large therapeutic doses.	Powerful for tracking macrophage-specific vesicles in co-culture systems, synovial fluid, or local callus samples.	[50]
Microfluidic-Based Isolation	Lab-on-a-chip microdevices integrating nano-scale filters, microchannels, or acoustic/electrical fields to sort and capture exosomes (size- or affinity-based)	Rapid processing (minutes) and small sample requirement; can combine isolation with on-chip detection/analysis (toward point-of-care diagnostics); diverse techniques (size-sieving, deterministic lateral displacement, acoustic wave separation, on-chip immunocapture) offer flexible design	Poor scalability – current devices handle only µL–mL volumes; requires specialized microfabricated chips and instruments; still in developmental stage for EV isolation (not standardized).	Diagnostic use – Promising for clinical analysis of exosomes (e.g. rapid isolation for biomarkers) but not yet suitable for therapeutic exosome manufacturing	Useful for monitoring dynamic M-Exo subpopulations in small volumes of fracture exudate or callus lavage fluid.	[54]

Different isolation methods can influence vesicle yield, purity, integrity, and subpopulation composition. Differential ultracentrifugation and density-gradient ultracentrifugation remain widely used in experimental studies, but they are time-consuming, difficult to scale, and may co-isolate protein aggregates or other contaminants [50]. Size-exclusion chromatography (SEC) and tangential flow filtration (TFF) offer gentler and more scalable alternatives, although they often require additional concentration or purification steps [54]. Immunoaffinity capture and microfluidic platforms can enrich specific vesicle subsets with higher specificity [53, 55], but their recovery rate, cost, and scalability remain important limitations [56]. These methodological differences are particularly relevant for M-Exos, because isolation bias may alter the apparent relationship between macrophage state, exosomal cargo, and downstream immunomodulatory function.

After isolation, M-Exos should be characterized using complementary physical, molecular, and functional assays. Particle size and concentration are commonly assessed by nanoparticle tracking analysis (NTA), while transmission electron microscopy (TEM) confirms vesicle morphology and helps identify co-isolated contaminants [57]. Molecular characterization should include positive exosomal markers such as CD9, CD63, CD81, ALIX, and TSG101, together with negative markers from non-vesicular cellular compartments. In addition, cargo profiling of regulatory RNAs, proteins, cytokine-associated signals, and lipids can help define the functional signature of M-Exos. Any macrophage-polarizing agents or preconditioning stimuli, such as LPS or IL-4, should be carefully removed before exosome isolation to avoid confounding effects from residual stimulants [58].

Overall, robust M-Exos characterization should move beyond basic vesicle identification toward an integrated quality framework that includes identity, purity, cargo composition, biological potency, and macrophage-state specificity. Functional potency assays, such as macrophage repolarization, MSC osteogenic differentiation, endothelial tube formation, or osteoclastogenesis inhibition, will be essential for linking vesicle properties to stage-specific immunomodulatory outcomes in bone regeneration. This integrated approach is particularly important for translating M-Exos from experimental vesicle preparations into reproducible and clinically regulatable regenerative therapeutics.

## Spatiotemporal immunomodulation and underlying mechanisms of M-Exos-mediated bone healing stages

**Fig. 2:**
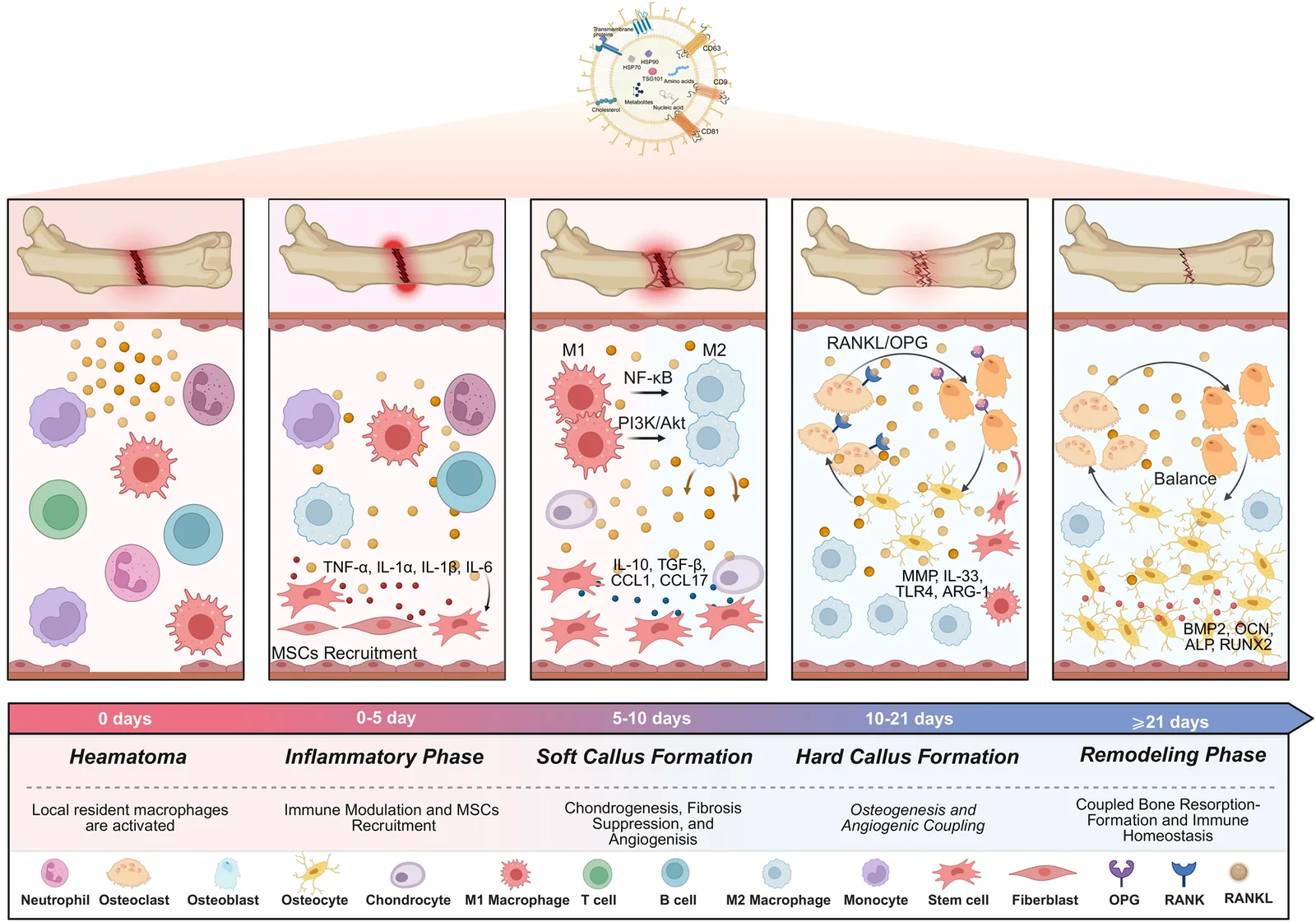
Spatiotemporal roles of M-Exos during sequential stages of bone healing. This schematic summarizes the stage-dependent actions of M-Exos within the multicellular bone- healing microenvironment, including hematoma formation at Day 0, the inflammatory phase at Days 0–5, soft callus formation at Days 5–10, hard callus formation at Days 10–21, and remodeling at Day 21 and beyond. In the acute inflammatory phase, M-Exos mediate inflammation resolution and progenitor cell recruitment to the fracture site. As the soft callus forms, they drive chondrogenesis and angiogenesis, establishing a vascularized cartilaginous matrix. During hard callus formation, M-Exos enhance osteoblastic differentiation while restraining premature osteoclastogenesis, promoting coordinated bone mineralization and vascular integration. In the remodeling phase, M-Exos sustain osteoclast-osteoblast coupling and an anti-inflammatory milieu, ensuring balanced resorption and mature bone architecture restoration. This phase-specific modulation underscores M-Exos as versatile regulators bridging immune crosstalk and regenerative cascades. (abbreviations: M-Exos, macrophage-derived exosomes; M1, classically activated macrophage; M2, alternatively activated macrophage; M1-exos, M1 macrophage-derived exosomes; M2-exos, M2 macrophage-derived exosomes; MSCs, mesenchymal stem cells; BMSCs, bone marrow mesenchymal stem cells; NF-κB, nuclear factor kappa B; PI3K, phosphoinositide 3-kinase; akt, protein kinase B; TNF-α, tumor necrosis factor alpha; IL, interleukin; TGF-β1, transforming growth factor beta; CCL, C-C motif chemokine ligand; RANK, receptor activator of NF-κB; RANKL, receptor activator of NF-κB ligand; OPG, osteoprotegerin; MMP, matrix metalloproteinase; TLR4, toll-like receptor 4; ARG-1, arginase-1; BMP2, bone morphogenetic protein 2; OCN, osteocalcin; ALP, alkaline phosphatase; RUNX2, runt-related transcription factor 2.)

Bone regeneration following fracture proceeds through highly coordinated, temporally overlapping stages, including the inflammation, soft callus formation, and remodeling [59]. Recent single-cell, spatial transcriptomic, and multimodal datasets have introduced a state-resolved layer to this classical temporal framework, revealing that macrophage populations are dynamically recruited and functionally diversified within the fracture-healing niche to coordinate complex multicellular crosstalk [60, 61]. Given that exosome biogenesis and cargo sorting are strictly governed by the donor macrophage’s functional state, metabolic activation, and local microenvironmental cues, M-Exos serve as critical vesicular intermediates that translate cellular programs into broader niche responses [34]. Consequently, M-Exos represent state-dependent vesicular outputs that mirror and transmit local macrophage dynamics rather than functioning as uniform, static vesicle preparations [62]. Within this paradigm, while single-cell RNA sequencing (scRNA-seq) does not directly profile exosomal cargo, it provides a rational blueprint to delineate donor macrophage states—offering an instructive roadmap to avoid inflammatory-risk modules and enrich pro-resolving or pro-regenerative components when engineering immunomodulatory M-Exos. Building upon these insights, this section maps the phase-specific mechanisms and spatiotemporal immunomodulatory landscapes through which M-Exos regulate bone healing (Fig. 2), while highlighting their translational utility in bone repair strategies.

### Acute inflammatory phase (days 0–5): immune modulation and MSC recruitment

The inflammatory phase is initiated immediately after fracture and is characterized by hematoma formation, neutrophil infiltration, and the rapid release of pro-inflammatory mediators, including TNF-α, IL-1β, and IL-6, within the injured microenvironment. During this early stage, tissue-resident macrophages and recruited monocyte-derived macrophages participate in debris clearance, matrix turnover, host defense, and initiation of repair [63]. In this context, M-Exos act as vesicular mediators of macrophage–microenvironment communication, contributing to inflammatory resolution and helping establish a regenerative niche that supports bone healing.

**M-Exos-Mediated Macrophage Reprogramming:** During the early inflammatory phase, M-Exos function as temporal regulators of inflammatory amplitude and resolution rather than as simple mediators of a linear M1-to-M2 switch. Vesicles released from inflammatory macrophage states may contribute to early immune activation by transmitting NF-κB/TLR-associated cues and cytokine-inducing cargo. However, when these inflammatory-state vesicles persist beyond the early reparative window, they may sustain excessive cytokine production, compromise progenitor-cell survival, and delay callus maturation. Consistent with this concept, M1-derived exosomes enriched in miR-155 have been shown to amplify inflammatory signaling in injury models, supporting the view that prolonged pro-inflammatory vesicular cargo can aggravate tissue damage when not temporally restrained [64].

By contrast, pro-resolving M-Exos actively constrain excessive inflammation and help establish a regenerative osteoimmune niche. IL-4-polarized M2-Exos carry regulatory microRNAs, including miR-99a, miR-146b, and miR-378a, which suppress NF-κB and TNF-α signaling and reduce inflammatory cytokine release [65]. Similarly, M2-derived exosomal miR-26b-5p promotes macrophage reprogramming by targeting the TLR3/NF-κB pathway [66], whereas miR-146a attenuates inflammatory signal transduction by inhibiting upstream NF-κB activators such as IRAK1 and TRAF6 [67]. These findings indicate that the immunomodulatory activity of M-Exos is encoded in vesicle-specific cargo programs that translate donor macrophage states into functional signals in recipient macrophages, stromal cells, and skeletal progenitors. Consequently, M-Exos provide a mechanistic bridge between macrophage-state dynamics and exosome-mediated immunomodulation, linking donor-cell activation, cargo sorting, recipient-cell reprogramming, and phase-specific regenerative outcomes within the bone-healing cascade.

**Progenitor Cell Recruitment and Osteogenic Priming:** In parallel with immune modulation, M-Exos directly influence skeletal progenitor behavior during the early fracture response. Liu et al. reported that exosomes from M1 macrophages are enriched in miR-21-5p and can transfer this microRNA to bone marrow mesenchymal stem cells, thereby promoting osteogenic differentiation [68]. MiR-21-5p has been associated with the suppression of osteogenesis-inhibitory pathways, including SMAD7 and PTEN signaling, leading to increased Runx2 expression and osteoblast-lineage commitment [69, 70]. These observations indicate that early inflammatory M1-Exos may not be uniformly detrimental; rather, when temporally restricted, they may provide an initial osteogenic-priming signal for recruited MSCs. Pro-resolving M-Exos may further support progenitor-cell recruitment and survival by creating a more permissive immune microenvironment. They have been shown to stimulate MSC migration, proliferation, and survival, while also promoting endothelial-cell recruitment for early neovascularization [71]. In an engineered scaffold model, Jin et al. demonstrated that electrospun scaffolds functionalized with M2-Exos markedly enhanced MSC migration through activation of the Rap1/PI3K/Akt pathway [72]. These exosomes also promoted anti-inflammatory macrophage polarization, supporting a feed-forward osteoimmune loop in which M2-Exos recruit reparative progenitors while simultaneously reinforcing immune resolution. Through these coordinated effects, early M-Exos may link macrophage-mediated immunomodulation with MSC recruitment and osteogenic priming, thereby bridging the inflammatory phase with the subsequent reparative phase [73].

**Angiogenic Stimulation:** Along with MSC recruitment, early M-Exos stimulate angiogenesis to vascularize the callus. Wang et al. observed that M2-Exos in a diabetic fracture model not only shifted macrophages toward a healing phenotype but also increased local capillary density and blood perfusion, partly by inducing pro-angiogenic factors [74]. In general, M-Exos carry proteins such as pro-angiogenic cytokines and RNAs that induce endothelial cells to form new blood vessels, ensuring adequate oxygen and nutrient supply to the healing tissue. Some studies have even loaded exosomes with VEGF-encoding plasmid cargo: for instance, progenitor cell-derived exosomes augmented with VEGF plasmids achieved robust vascular remodeling and bone union in critical defects [40]. Naturally secreted M-Exos achieve a similar outcome by upregulating endogenous VEGF and other angiogenic pathways in the fracture microenvironment. The result is the formation of a well-vascularized granulation tissue rich in MSCs, which is the hallmark of the late inflammatory/early repair phase.

**Early Extracellular Matrix (ECM) Remodeling:** Additionally, M-Exos contribute to extracellular matrix (ECM) remodeling in the fracture hematoma, which indirectly supports osteogenesis [75]. During days 0–5, the provisional matrix must be turned over and refined into a scaffold for new bone [76]. Macrophages and their exosomes stimulate surrounding stromal cells such as fibroblasts, periosteal cells, and MSCs to produce collagen and osteoid and also secrete matrix metalloproteinases to remove necrotic tissue [77]. These processes are partly driven by exosomal growth factors and enzymes that modulate the ECM composition. For example, M-Exos have been reported to carry lysyl oxidase and annexins that promote collagen cross-linking and mineral deposition in the matrix, thereby priming the site for callus formation [78]. By fine-tuning the inflammatory milieu and remodeling the ECM niche, M-Exos set the stage for indirect osteogenesis – they create a permissive environment in which recruited MSCs can survive, proliferate, and differentiate effectively.

In summary, during the early inflammatory phase of bone healing (day 0–5), M-Exos serve as critical communicators that link immune responses with regenerative outcomes. The concerted actions of M-Exos during the first few days post-injury illustrate a sophisticated form of “immune orchestration”: they transform the chaotic inflammatory surge into a structured reparative program, ultimately guiding MSCs and other cells toward bone regeneration [79]. This early crosstalk via exosomes is indispensable for successful fracture healing and represents a paradigm of the tight coupling between the immune system and tissue regeneration.

### Soft callus formation (days 5–10): chondrogenesis, anti-fibrotic control, and angiogenesis

As the inflammatory phase resolves, the fracture microenvironment shifts towards tissue formation [80]. The soft callus phase is characterized by the differentiation of progenitor cells within the hematoma into a soft cartilaginous callus that bridges the fracture gap. M-Exos play a pivotal role in orchestrating this transition by modulating stem cell behavior and tissue remodeling. Their ability to regulate mesenchymal progenitors, inhibit fibrotic responses, and promote neovascularization highlights their multifaceted contribution during the early reparative phase.

**Promoting Chondrogenesis:** During the soft callus phase, M-Exos actively foster chondrogenesis. Cai et al. demonstrated that M2-Exos can rejuvenate aged mesenchymal stem cells (MSCs), alleviating cellular senescence and enhancing their chondrogenic differentiation potential. In an aged rat tendon–bone healing model, local delivery of M2-Exos promoted the formation of a robust fibrocartilaginous interface, indicating accelerated cartilage matrix deposition [81]. Similarly, Qian et al. found that exosomal microRNA cargo from M2 macrophages can “re-educate” pro-inflammatory M1 macrophages toward an anti-inflammatory phenotype while directly inhibiting chondrocyte hypertrophy. These dual pro-chondrogenic and anti-catabolic effects are particularly valuable during the soft callus phase, where excessive hypertrophy or matrix degradation can compromise downstream endochondral ossification [66]. Notably, therapeutic application of M-Exos during this phase serves not only to direct MSC fate toward chondrogenesis but also to stabilize the developing callus microenvironment [82]. This ensures a favorable scaffold for mineralization and hard callus maturation in the later stages of fracture repair.

**Suppressing Fibrotic Scar Formation:** Unlike many tissues, bone heals without a permanent fibrotic scar – a feature partly attributed to timely macrophage signaling in the callus. M-Exos appear to contribute to this scar-free healing by modulating fibroplasia during the soft callus stage [83]. Linda Vi et al. observed that constitutive macrophage ablation leads to “failed” fracture repair characterized by small, fibrous calluses with scant bone. In their transgenic mouse model, fractured callus tissue contained excessive collagenous fibrotic tissue and insufficient bone when macrophage-derived signals were absent [84]. This pathological outcome resembles a fibrotic non-union, implying that normal macrophage activity and exosomal paracrine signaling are required to inhibit scar-like tissue deposition.

Consistent with this, immunomodulatory therapies that boost M2 macrophage activity in early fracture repair tend to reduce fibrous tissue and improve bone regeneration [85]. For example, Claudia Schlundt et al. induced M2 polarization in vivo via IL-4/IL-13 delivery and reported significantly enhanced endochondral bone formation with minimal fibrous tissue, highlighting that an anti-inflammatory macrophage milieu curbs fibrotic tissue infiltration in the callus [86]. By delivering anti-fibrotic factors such as IL-10 and TGF-β in exosomes, M2-like macrophages likely direct progenitor cells to form cartilage/bone rather than scar [39]. Indeed, reparative M2-Exos loaded with IL-10 mRNA have been shown to suppress pathological collagen deposition in inflammatory bone loss models [28]. Collectively, these findings suggest that M-Exos help “fine-tune” the healing environment to favor regenerative callus tissue over fibrotic scar, ensuring proper cartilage scaffold formation for bone healing.

**Supporting Angiogenesis:** Angiogenesis is critical in the soft callus as the nascent cartilage is vascularized and later mineralized. M-Exos actively support new vessel formation within the callus bridge. Wen et al. engineered a biomimetic periosteum by electrospinning a nanofiber scaffold loaded with M2-Exos, and observed markedly increased neovascularization and cell recruitment in segmental bone defects. The M2-Exos-rich scaffold released exosomes that stimulated endothelial cell migration and tube formation, ultimately leading to a higher density of CD31^+^ capillaries in the regenerating bone. These pro-angiogenic effects are attributed to exosomal growth factors and microRNAs that activate angiogenic pathways in endothelial cells [87]. In parallel, M-Exos can influence vascular supporting cells. Yan et al. reported that M2-Exos promote a reparative c-Kit^+^ phenotype in vascular smooth muscle cells, which in turn facilitates vessel remodeling after injury. By inducing c-Kit expression, M2-Exos helped convert contractile smooth muscle cells into a more stem-like, pro-angiogenic state during vascular repair. Extrapolated to fracture healing, this suggests M-Exos not only deliver pro-angiogenic signals to endothelial cells but also modulate perivascular cells to support neovessel integration into the callus [88]. Furthermore, recent evidence from osteonecrosis models shows that M2-Exos enriched with miR-93-5p can drive endothelial phenotypic changes that enhance angiogenesis [4]. In summary, M-Exos in the soft callus promote angiogenesis by providing molecular cues that stimulate neovascularization, thereby enhancing oxygen and nutrient delivery required for callus maturation.

**Coupling Inflammation Resolution with Repair:** Timely resolution of inflammation is tightly coupled with the progression of fracture repair – a process in which M-Exos play a pivotal coordinating role. For instance, Zhang et al. showed that exosomes from M1-like bone marrow macrophages in diabetic mice carry miR-144-5p, which blunts Smad1 signaling in MSCs and correlates with excess cartilaginous tissue and impaired bone formation in the callus [89]. Such findings underscore that if inflammatory macrophage exosomal signals persist, the soft callus may fail to mature properly and may manifest as enlarged cartilage and delayed mineralization. On the other hand, augmenting pro-resolving M-Exos can synchronize inflammation tapering with reparative activities. Wang et al. found that administering M2-Exos in a diabetic fracture model accelerated the switch from an M1-dominant milieu to an M2-rich one, thereby shortening the inflammatory phase and expediting callus development. Treated fractures showed earlier angiogenesis and ossification compared to controls, suggesting that M2-Exos helped “couple” immune resolution with tissue regeneration. Consistent with this, raising local IL-4/IL-10 levels at the fracture site is known to promote earlier remodeling of cartilage into bone [74]. Essentially, M-Exos serve as messengers that ensure the inflammatory phase winds down at the appropriate time, ceding the stage to chondrogenesis, angiogenesis, and osteogenesis in the soft callus.

Collectively, these findings underscore the multifaceted therapeutic potential of exogenously administered M-Exos during the soft callus stage of fracture healing. By delivering reparative molecular cues—including anti-senescent microRNAs, anti-fibrotic cytokines, and pro-angiogenic regulators—M-Exos actively reshape the transitional microenvironment from inflammation to regeneration. Their ability to rejuvenate MSCs and direct them toward a chondrogenic lineage, suppress fibrotic scarring, stimulate neovascularization, and synchronize immune resolution with tissue repair makes them central regulators of early-stage bone regeneration. Unlike endogenous exosomes that may be influenced by pathological states, externally delivered M-Exos offer precise temporal and phenotypic control, enabling tailored modulation of the local milieu. This well-structured soft callus, supported by exosomal coordination, forms the biological and mechanical foundation necessary for effective mineralization and the onset of hard callus formation.

### Hard callus formation (days 10–21): osteogenesis and angiogenic coupling

During the hard callus phase, the soft cartilaginous callus is gradually replaced by a mineralized bony callus through endochondral and intramembranous ossification, a process tightly coupled with neovascularization. Macrophage activity is critical in this stage; indeed, deficiencies in macrophages or their appropriate polarization can slow hard callus formation and extend healing time. Emerging evidence indicates that M-Exos serve as pivotal paracrine regulators in this phase, regulating osteogenic differentiation, bone matrix deposition, vascularization, and the timely recruitment of remodeling cells.

**Inducing Osteogenic Differentiation of MSCs/Osteoblast Precursors:** M-Exos potently induce osteogenic commitment of MSCs and osteoprogenitors. Xia et al. reported that exosomes from classically activated M1 macrophages markedly enhanced the osteogenic differentiation of bone marrow MSCs in vitro, with M1-Exos exerting a stronger pro-osteogenic effect than exosomes from unpolarized or M2 macrophages [90]. However, other studies have observed pro-osteogenic effects from both M1 and M2-Exos populations. Wang et al. identified a specific long non-coding RNA (lncRNA, LOC103691165) enriched at similar levels in exosomes from both M1 and M2 polarized macrophages that significantly promote MSC osteogenesis [91]. Likewise, Luo et al. demonstrated that exosomes from either M1 or M2 macrophages could drive murine MSCs toward an osteoblastic lineage, with M1-Exos particularly effective at the early inflammatory stage via transfer of miR-21a-5p, which upregulated the osteogenic transcription factor RUNX2 to accelerate early osteogenic commitment. These findings suggest that regardless of polarization state, macrophages secrete exosomal factors that prime MSCs for osteogenesis [92]. Notably, there may be temporal and contextual differences – M1-derived exosomes seem to provide an “early burst” to initiate osteogenesis via signals like miR-21a-5p during the initial inflammatory phase, whereas M2-derived exosomes sustain osteogenic induction during the later healing phase through other cargo.

**Enhancing Osteoblast Function and Matrix Mineralization:** Beyond lineage commitment, M-Exos can either support or hinder the function of osteoblasts in bone matrix formation, depending on their polarization and timing. Notably, the pro-osteogenic influence of M1-Exos appears limited to the early phase; if M1-Exos persist into the matrix-deposition stage, their inflammatory cargo may become deleterious to bone formation. Consistent with this, Miya et al. provided in vivo evidence that exosomes from pro-healing macrophages (M2/M0) significantly promoted new bone regeneration in a rat calvarial defect model, whereas sustained M1-Exo exposure impaired bone repair. Thus, timing and context are critical in reconciling these divergent findings. M1-Exos may deliver an initial osteogenic spark during the inflammatory phase, but effective healing requires a transition to M2-Exos-dominated, anti-inflammatory signals to sustain bone formation once the acute phase resolves. Mechanistically, differences in M-Exos cargo help explain this dichotomy. Kang et al. found that M1-Exos were enriched in miR-155, which impairs osteoblast activity by downregulating pro-osteogenic signals such as BMP2/BMP9, whereas M2-Exos contained miR-378a, which stimulates osteoblasts via the BMP pathway [41]. Altogether, these findings indicate that appropriately polarized M-Exos markedly potentiate osteoblast function and matrix mineralization, whereas an unmodulated M1 exosomal profile can instead exacerbate inflammation and inhibit those processes [94].

**Orchestrating Angiogenesis and Coupling with Osteogenesis:** Angiogenesis is tightly coupled to osteogenesis during hard callus formation, as new blood vessel ingrowth is required to supply oxygen, nutrients, and osteoprogenitor cells to the developing bone. M-Exos play a key role in coordinating this angiogenic–osteogenic coupling. Anti-inflammatory M2 macrophages are well-known producers of pro-angiogenic factors, and their exosomes similarly carry signals that stimulate blood vessel formation in the callus [95]. Yang et al. demonstrated that exosomes from M2 macrophages deliver microRNA-93-5p to endothelial cells, thereby suppressing pathological neutrophil extracellular traps (NETs) and preventing endothelial-to-mesenchymal transition, while upregulating VEGF expression in local endothelial cells. In a bone injury model of osteonecrosis, these M2-Exos enriched with miR-93-5p markedly enhanced microvascular formation and perfusion, which in turn supported new bone tissue regeneration. This underscores how M-Exos foster a regenerative vascular niche within the callus [4]. In related work, Guo et al. reported that M2-Exos promoted angiogenesis in ischemic cardiac tissue by transferring miR-132-3p to endothelial cells; miR-132-3p in exosomes targeted thrombospondin-1 (THBS1), thereby unleashing pro-angiogenic responses [96]. By analogy in bone healing, M-Exos likely deploy similar pro-angiogenic miRNAs such as miR-132-3p to stimulate endothelial cell migration, sprouting, and network formation in the fracture callus. In diabetic fracture healing where angiogenesis is often impaired, the administration of M2-Exos has been shown to increase capillary density at the fracture site, coupling improved blood supply with accelerated bone formation [74]. Thus, M-Exos serve as mediators of angiogenic–osteogenic coupling, ensuring that nascent bone formation in the hard callus is supported by adequate vascularization.

**Modulating Osteoclast Recruitment and Activity:** During the hard callus phase of bone healing, timely and spatially controlled osteoclast recruitment is essential for transitioning the mineralized callus into mature lamellar bone. M-Exos play a dual role in orchestrating this process: they not only modulate the migration of osteoclast precursors but also influence their subsequent differentiation [97]. Yuan and Li’s review suggests that infiltrating M1 macrophages in arthritic bone disease contexts promote osteoclast formation by secreting IL-6 and TNF-α, whereas M2-Exos may carry factors that counteract this osteoclastogenic milieu [93]. In the fracture callus, this balance ensures that osteoclasts are recruited at the right time and in the right numbers – enough to remodel the hard callus later, but not so early or numerous as to destabilize the repair. In addition to modulating recruitment, M-Exos directly influence osteoclast differentiation pathways, often exerting an inhibitory effect on osteoclastogenesis during the hard callus stage [98]. Exosomes from M2 macrophages have been shown to suppress RANKL-induced osteoclast differentiation. Chen et al. found that M2-Exos significantly inhibited osteoclast formation in vitro, as evidenced by fewer TRAP-positive multinucleated cells. They identified exosomal microRNA-1227-5p as a key mediator: M2-Exos transfer miR-1227-5p into osteoclast precursors, where it targets and downregulates OSCAR (osteoclast-associated receptor), a co-stimulatory molecule essential for osteoclastogenesis. Inhibition of miR-1227-5p reversed the anti-osteoclastogenic effect, confirming that M2 exosomal cargo can intrinsically block osteoclast differentiation pathways [99]. Similarly, other studies report that M2-Exos deliver proteins that interfere with pro-osteoclast signaling; for example, exosomal transfer of the deubiquitinase CYLD into osteoclast precursors inactivates STAT3 – a transcription factor that drives osteoclast gene expression – thereby curbing osteoclastogenesis [30]. Notably, exosomal CYLD can also attenuate NF-κB signaling in osteoclasts, broadening its inhibitory impact on bone resorption [100]. Through these mechanisms, M-Exos help preserve the hard callus from premature degradation, maintaining structural continuity until remodeling can proceed in a physiologically coordinated manner.

### Remodeling phase (days 21+): coordinating resorption–formation balance and immunoregulation

In the late remodeling phase of fracture healing, the immature woven bone of the hard callus is progressively resorbed and replaced by mature lamellar bone, ultimately restoring the original architecture and mechanical strength of the skeleton. This phase is defined by tightly coordinated cycles of osteoclast-mediated resorption and osteoblast-driven formation. Therefore, late-stage M-Exos should be interpreted primarily as coupling regulators that terminate residual inflammation, restrain excessive osteoclastogenesis, support osteoblast-lineage activity and preserve long-term osteoimmune homeostasis, rather than as generic pro-osteogenic stimuli. This framing directly links immunomodulation to vesicle biology: the relevant question is which donor macrophage state generates vesicular cargo capable of restoring resorption-formation balance in the remodeling niche.

**Regulating Osteoclastogenesis and Bone Resorption:** During remodeling, M-Exos continue to inhibit osteoclastogenesis to prevent excessive resorption. Exosomes from IL-4–polarized macrophages, for instance, suppress RANKL-driven osteoclast differentiation by downregulating key cytokines (GM-CSF, TNF-α) and co-stimulatory receptors (like OSCAR) in osteoclast precursors [98]. Mechanistically, M-Exos deliver specific anti-osteoclastogenic cargo – including anti-inflammatory cytokines and microRNAs – that converge on osteoclastogenic pathways such as NF-κB/STAT3 and RANKL–NFATc1 signaling to restrain bone resorption in the remodeling phase. By tempering osteoclast formation and activity, M-Exos prevent excessive callus degradation, ensuring that resorption proceeds in balance with new bone formation.

**Supporting Osteoblasts and Osteocytes:** M-Exos also support fracture remodeling by influencing osteoblast differentiation, function, and the osteocyte network. A growing body of work shows that both classically activated (M1) and alternatively activated (M2) macrophage exosomes can enhance osteoblastogenesis from mesenchymal stem cells (MSCs). Liu et al. found that co-culture with M-Exos promoted the osteogenic commitment of bone marrow MSCs, with M1-Exos accelerating early osteogenic differentiation via exosomal microRNA-21a-5p, and M2-Exos sustaining longer-term osteogenesis [37]. However, inconsistencies in the pro-osteogenic effects of M1- and M2-derived exosomes across studies likely reflect differences in experimental conditions, timing, donor macrophage state, and recipient-cell context. These variables align with single-cell evidence that macrophage and hematopoietic populations occupy discrete but plastic states rather than fixed binary identities. As healing progresses, M2-Exos become dominant and deliver pro-osteogenic signals. Liu et al. demonstrated that M2-Exos transfer miR-22-3p to MSCs, downregulating the Wnt inhibitor PER2 and thereby unleashing Wnt/β-catenin signaling to enhance osteoblast differentiation [101]. Similarly, Liu et al. showed that M2 exosomal miR-486-5p directed MSCs toward the osteoblast lineage via the TGF-β1/Smad2 pathway and even rescued bone loss in osteoporotic mice [102]. Collectively, these findings indicate that M-Exos establish a microenvironment rich in osteogenic cues, facilitating osteoblast maturation and bone matrix production. Moreover, by fostering a well-perfused and anti-inflammatory niche, M-Exos likely aid in preserving the viability of nascent osteocytes formed during this phase. VEGF induced by M-Exos not only couples angiogenesis with osteogenesis but also directly preserves osteocyte survival, suggesting that the exosomal signaling environment helps maintain a healthy osteocyte network in the remodeled bone [103].

**Coupling Formation and Resorption:** Bone remodeling is a tightly coupled process in which osteoclast-mediated resorption and osteoblast-mediated formation are spatiotemporally coordinated. Bone-resident macrophages, particularly osteomacs, are crucial regulators within this process. They localize on bone surfaces and secrete factors such as TGF-β1 and BMP-2, attracting osteoblast precursors to sites of recent bone resorption [104]. Weivoda et al. emphasized the central role of macrophages in orchestrating bone remodeling by releasing cytokines and exosomes, thus ensuring balanced osteoclast and osteoblast activity [105]. Recent insights from Sun et al. and Behera et al. highlight exosomal signaling as an essential mechanism for coupling crosstalk between bone cells [106, 107]. M-Exos simultaneously limit excessive osteoclastogenesis and stimulate osteogenesis by transferring bioactive molecules, such as miRNAs and proteins, to target cells. These exosomes thus ensure that new bone formation closely follows bone resorption, resulting in a well-vascularized and effectively remodeled bone structure. Consequently, M-Exos serve as critical coupling mediators, coordinating the precise timing and magnitude of bone resorption and formation. Their role ensures osteoclasts and osteoblasts function in harmony, preventing pathological bone resorption or unchecked bone formation, which is vital for restoring normal bone architecture during the remodeling phase.

**Maintaining an Anti-Inflammatory Environment:** The transition from inflammation to immune resolution during fracture healing is largely governed by macrophages, with exosome-mediated signaling playing an integral role in this process. M-Exos help maintain this pro-healing, anti-inflammatory environment by reprogramming immune cells and dampening inflammatory signaling. Wang et al. demonstrated that M2-Exos act as potent immunomodulators in diabetic fracture models: administration of M2-Exos significantly decreased M1 macrophage proportions in the callus, increased the relative abundance of M2 macrophages, and lowered expression of inflammatory cytokines like TNF-α and IL-1β. Mechanistically, these exosomes induced M1-to-M2 phenotypic switching via activation of the PI3K/Akt pathway in target cells [74]. The net effect is a resolution of inflammation – exosomal cargo such as IL-10, TGF-β1, and anti-inflammatory microRNAs suppress pro-inflammatory mediator production while boosting anti-inflammatory signals. In addition to macrophages themselves, other immune and stromal cells are influenced by M-Exos: studies have noted that M-Exos can promote regulatory T-cell development and inhibit Th1/Th17 responses that drive osteoclast activation [108]. Single-cell-defined inflammatory and reparative gene modules may therefore serve as potency benchmarks for engineered M-Exos, allowing future products to be evaluated not only by particle number or canonical EV marker expression but also by their ability to suppress inflammatory macrophage programs and enrich pro-resolving, angiogenic communication modules.

**Summary:** Bone regeneration requires the coordinated resolution of inflammation, recruitment of progenitor cells, extracellular matrix remodeling, angiogenesis, osteogenesis, and the remodeling of immature callus into mature bone. Throughout this process, M-Exos act as state-dependent vesicular mediators that transmit macrophage-derived regulatory cues to immune, stromal, endothelial, osteogenic, and osteoclastic cells. Their biological effects depend not only on intrinsic vesicle activity, but also on the donor macrophage state, cargo composition, and the timing and location of vesicle delivery within the repair microenvironment.

This stage-dependent mode of action supports the use of M-Exos as cell-free osteoimmunomodulatory agents for bone regeneration, particularly in settings where endogenous repair is compromised, such as aging, diabetes, inflammatory bone loss, and critical-sized defects. Further translation should therefore focus on controlling donor-cell programming, cargo selection, targeting specificity, and local retention during production and delivery. Surface modification, cargo enrichment, donor-cell preconditioning, and biomaterial-assisted delivery provide practical routes to improve the reproducibility and stage specificity of M-Exo-based regenerative therapies

## Bioengineering strategies for M-Exos: surface modification, cargo enrichment, and biomaterial-driven delivery

Having outlined the stage-dependent roles of M-Exos in bone healing, the central translational question is how these vesicles can be engineered into targeted, reproducible, and therapeutically potent regenerative products. Current engineering efforts therefore focus on controlling vesicle biodistribution, cargo composition, and residence time within the repair microenvironment [109]. Major strategies include surface modification to enhance bone tropism, donor-cell preconditioning and genetic manipulation to tune endogenous cargo, exogenous cargo loading to increase therapeutic potency, and biomaterial-assisted delivery to prolong local retention.

This section, we discuss these strategies, representative recent advances, and their translational prospects.

### Surface engineering for enhanced bone - specific targeting

Ensuring that systemically delivered exosomes reach an injured bone site in sufficient quantity remains a major challenge in exosome therapy. To overcome this hurdle, researchers have developed various surface engineering strategies that functionalize exosome membranes with targeting ligands, thereby conferring bone-homing specificity and overcoming the nonspecific biodistribution of unmodified vesicles. Broadly, these approaches include the attachment of bone-seeking peptides, glycoprotein ligands, nucleic acid aptamers, bone-affinitive small molecules, or even chemokine receptors to the exosome surface, all aimed at directing the vesicles to bone tissue.

One strategy is to functionalize exosomes with osteotropic peptides or protein motifs that recognize components of bone or the bone marrow microenvironment. For example, polyaspartate-rich peptides (Asp-Ser-Ser, DSS) have a strong affinity for hydroxyapatite in the bone matrix. Exosomes displaying multimeric DSS peptides have shown enhanced binding to bone and improved delivery of therapeutic payloads to skeletal lesions in preclinical models. Similarly, glycoengineering the exosomal surface to present bone marrow homing ligands can facilitate uptake by bone niche cells. E-selectin on bone marrow endothelium is a key adhesion receptor for hematopoietic stem cell homing; accordingly, decorating exosome membranes with E-selectin–binding glycoproteins (GLG1, E-selectin ligand-1) markedly increases their accumulation in bone marrow [110].

**Fig. 3:**
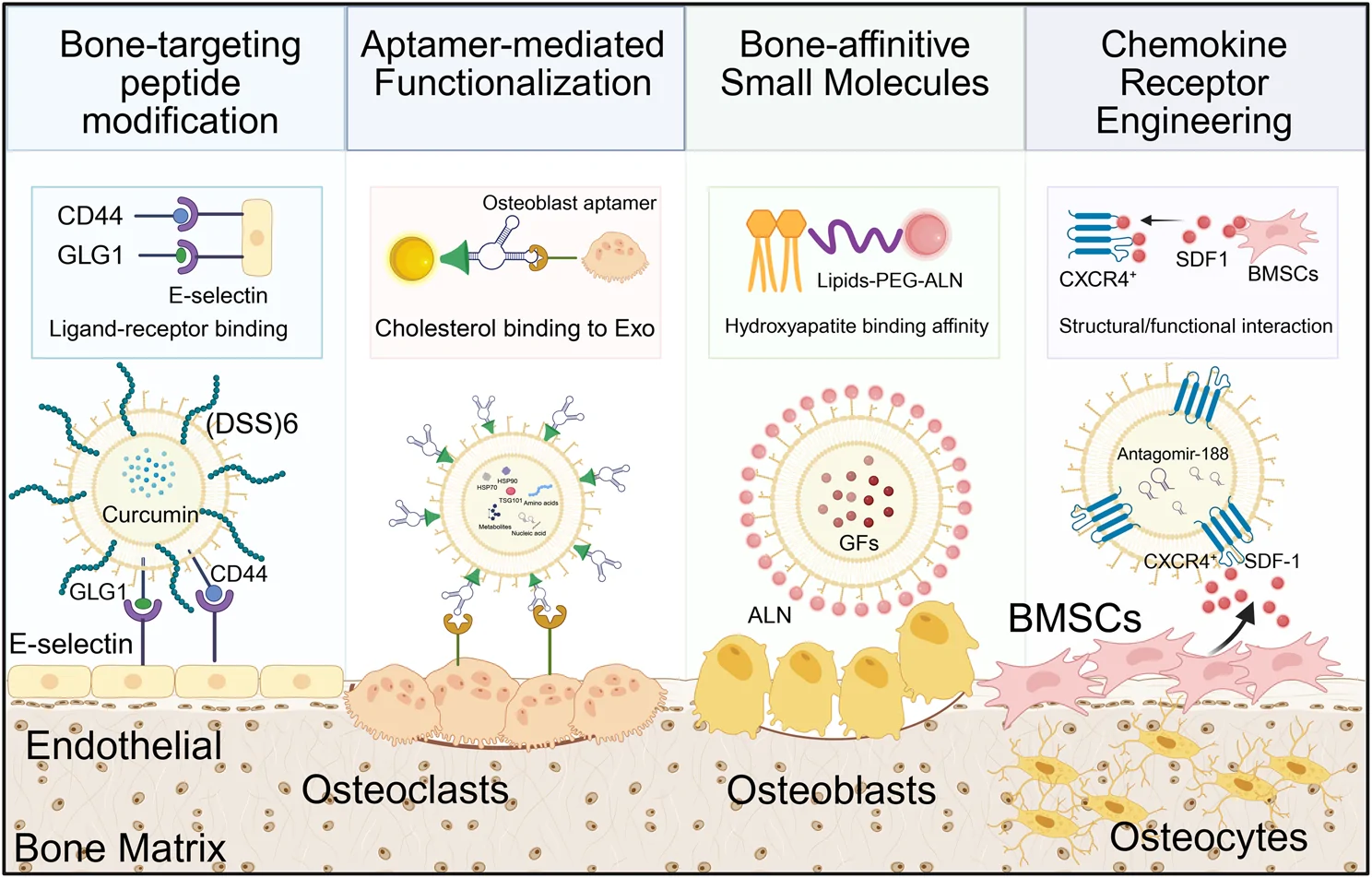
Advanced engineering strategies for bone-targeted M-Exos delivery. Conceptual overview of bioengineering strategies to optimize the targeted delivery and regenerative efficacy of M-Exos in bone repair. Key tactics include exosomal surface modification with bone-affinity ligands (e.g., collagen-binding peptides, aptamers, or bisphosphonate conjugates) to enable site-specific homing post-systemic administration. Complementary methods involve genetic manipulation or preconditioning of donor macrophages to augment exosomal cargo with osteoinductive elements (e.g., pro-osteogenic microRNAs or proteins). These integrated modifications enhance M-Exos accumulation at bone defects, amplify their immunomodulatory and osteogenic signaling, and hold promise for overcoming translational barriers in clinical bone tissue engineering

Another promising approach is aptamer-mediated targeting of exosomes. Nucleic acid aptamers that bind specifically to bone cell surface markers can be anchored onto exosomal membranes to serve as molecular “zip codes” for bone tissue. Recent studies have demonstrated that exosomes decorated with a BMSC-specific aptamer preferentially accumulate in bone and enhance therapeutic efficacy. For instance, Shou et al. reported that an RNA three-way junction nanoparticle bearing a BMSC-targeting aptamer could be used to functionalize M2-Exos. These aptamer-equipped exosomes homed to BMSCs in a mouse femur fracture model, resulting in significantly greater localization at the fracture site and markedly improved osteogenesis and healing compared to unmodified exosomes [111]. Similarly, Luo et al. showed that exosomes conjugated with a BMSC-selective aptamer exhibited enhanced homing to the bone marrow, which led to increased bone formation in osteoporotic mice and accelerated fracture repair in a femoral defect model. Such findings establish aptamer functionalization as an effective strategy to direct exosomes to specific bone cell populations [112]. A third category of surface engineering exploits bone-affinitive small molecules such as bisphosphonates to guide exosomes to bone. Alendronate (ALN), a bisphosphonate drug with high binding affinity for calcium-rich bone mineral, has been widely used as a bone-targeting moiety. By chemically conjugating ALN to exosomal membrane components via bio-orthogonal click chemistry, researchers have created ALN-functionalized exosomes that avidly bind to hydroxyapatite on bone surfaces. Wang et al. demonstrated that such ALN-tagged exosomes exhibit substantially greater hydroxyapatite binding in vitro and higher accumulation in bone in vivo, compared to unmodified exosomes [113]. In preclinical osteoporosis models, systemically administered ALN-conjugated exosomes homed to resorptive bone lesions and contributed to restoring bone density, illustrating the translational potential of bisphosphonate-guided exosome delivery for treating bone loss. Yet another strategy is chemokine receptor engineering to harness natural homing pathways. In this approach, exosome-producing cells are genetically engineered to overexpress certain chemokine receptors on the exosomal surface, so that the released vesicles will follow physiological chemotactic gradients. A prominent example is the SDF-1/CXCL12–CXCR4 axis involved in bone marrow homing. CXCR4 is the receptor that hematopoietic stem cells use to migrate to SDF-1–rich niches in bone marrow [114]. By endowing exosomes with CXCR4 on their surface, they can similarly sense the SDF-1 gradient and home to bone marrow. Hu et al. demonstrated that exosomes displaying CXCR4, when injected intravenously, selectively accumulated in bones where SDF-1 levels are high. These CXCR4+ exosomes effectively engrafted into the bone marrow compartment. Furthermore, when loaded with a therapeutic microRNA inhibitor (antagomir-188) and administered in an osteoporotic mouse model, the CXCR4-engineered exosomes specifically homed to the skeletal tissue and released their cargo, ultimately promoting new bone formation and reversing bone loss in vivo [115]. Beyond these biological ligand-based techniques, several physical targeting methods are being explored to improve exosome homing to bone. One example is magnetic targeting: loading exosomes with superparamagnetic iron oxide nanoparticles allows the vesicles to be guided to a desired bone region using an external magnetic field [116]. Magnetically driven targeting has already been tested in other tissue contexts and could similarly enhance the localization of exosomes to skeletal injury sites in a noninvasive manner. Another adjunct strategy is to prolong exosome circulation time by presenting “don’t-eat-me” signals on the vesicle surface. For instance, displaying the macrophage-inhibitory protein CD47 on exosomes can reduce their uptake by phagocytes in the liver and spleen, thereby reducing premature clearance and allowing more time for the exosomes to reach bone tissue [117]. There is also growing interest in using focused ultrasound to improve targeted delivery. Ultrasound can transiently increase local vascular permeability or even trigger the release of drugs at a specific site [118]. In this context, ultrasound-targeted microbubble destruction has been shown to enhance the tissue-specific delivery of therapeutic exosomes in other disease models [119], and a similar approach could be adapted to concentrate exosomes in a fracture or bone defect area. Overall, these surface-engineering innovations address a critical translational challenge for exosome therapy, as illustrated in Fig. 3, by dramatically increasing the concentration of bioactive vesicles at the target bone site. By improving homing specificity and bioavailability in the bone microenvironment while minimizing off-target distribution, such strategies promise to enhance the efficacy of exosome-based treatments for bone repair and regeneration.

### Advanced strategies for M-Exos cargo loading: endogenous and exogenous approaches

M-Exos have emerged as instructive carriers for bone repair because they couple a membrane context that supports tissue tropism and cellular uptake with a multi-layered cargo (miRNAs, mRNAs, proteins and lipids) capable of coordinating osteogenesis, angiogenesis and immunomodulation. However, the native payload of unmodified vesicles is often insufficient to achieve robust efficacy across clinically relevant defect sizes and hostile inflammatory microenvironments. Consequently, two broad engineering paradigms have been pursued to increase therapeutic “information density” in M-Exos: endogenous loading, which reprograms donor cells so that desired molecules are incorporated during exosome biogenesis, and exogenous loading, which introduces cargo into purified exosomes after isolation using physical or chemical perturbations of the vesicle membrane [120].

#### Endogenous loading approaches

Endogenous loading leverages the cell’s own machinery to package therapeutic cargo into exosomes. One route is genetic enrichment, whereby donor cells are transfected or otherwise engineered to overexpress a target gene, mRNA, or microRNA so that their secreted exosomes are enriched with that payload. Several studies underscore the dramatic impact genetic cargo enrichment can have on bone regeneration. Luo et al. showed that exosomes from M1 macrophages overexpressing miR-21a-5p accelerated mesenchymal stem cell differentiation and bone defect healing in vivo [68]. In one study, miR-365–2-5p-enriched M2-Exos promoted osteoblast differentiation by targeting and suppressing an inhibitory gene (OLFML1) in those cells [94]. Beyond miRNAs, other genetic cargos and signaling proteins have been loaded into exosomes to create more therapeutically potent vesicles. For instance, M2-Exos naturally carry the anti-inflammatory cytokine IL-10; by increasing the IL-10 content in these exosomes, researchers can concurrently promote osteoblast activity and inhibit osteoclast-mediated bone resorption. In a bone loss model of periodontitis, IL-10–enriched M2-Exos strongly activated the IL-10/IL-10 R signaling pathway in recipient cells, which protected alveolar bone from resorption while also aiding repair [39]. Similarly, loading exosomes with osteogenic mRNAs has shown great promise. Yang et al. engineered donor cells with a plasmid construct so that their exosomes contained high levels of BMP-2 mRNA; when these gene-augmented exosomes were embedded in a hydrogel and applied to a bone injury site, they continuously released BMP-2 signals and dramatically enhanced new bone formation in a critical-size calvarial defect [121]. Investigators are even exploring the inclusion of whole therapeutic pathways – for example, delivering CRISPR-Cas9 components or other gene editors via exosomes [122] – to stimulate osteogenesis at a genetic level, though such techniques are still in early stages.

Beyond genetic manipulation, endogenous cargo enrichment can be achieved by donor-cell preconditioning, exploiting the tight coupling between macrophage state and exosome biogenesis to bias vesicular cargo selection. Defined biochemical or biophysical cues can rewire inflammatory–metabolic programs and, in turn, remodel the exosomal proteome and transcriptome; for example, priming M2 macrophages with the slow-release H₂S donor GYY4137 yields exosomes with enhanced bone-regenerative activity, consistent with enrichment of uptake-facilitating components such as moesin that improve delivery to recipient MSCs [123]. Importantly, because preconditioning avoids permanent genetic modification, it may represent a more scalable and regulatory-tractable route to manufacturing potency-enhanced M-Exos. Low oxygen culture mimics the ischemic environment of healing bone fractures and has been shown to spur macrophages to release exosomes enriched in pro-angiogenic factors like VEGF, which could aid the vascularization of regenerating bone [35]. Mild inflammatory priming can also paradoxically boost certain growth factors in exosomes – for example, brief exposure of macrophages to a Toll-like receptor agonist might induce them to secrete vesicles that later help dampen inflammation and encourage repair. Even mechanical cues have been tested: subjecting macrophages to fluid shear stress or exposing them to biochemical signals from exercising muscle tissue has yielded exosomes that promote osteogenesis [124]. In summary, endogenous loading biologically “coaxes” the donor cells into making more potent exosomes. This strategy takes advantage of the cell’s own packaging mechanisms to ensure that the vesicles carry a higher concentration of the desired therapeutic cargo in a physiologically relevant manner.

#### Exogenous loading approaches

Exogenous loading strategies bypass the need to alter donor cells by instead loading therapeutic cargo directly into exosomes after they have been isolated. In this approach, purified exosome preparations are mixed with the cargo of interest, and then a physical or chemical technique is applied to facilitate the cargo’s entry across the vesicle membrane. A variety of methods have been developed to achieve this, the most common being electroporation and sonication. In electroporation, an electrical field is used to transiently open nanopores in the exosome membrane, allowing cargo (for example, siRNA, mRNA, or drug molecules) to diffuse inside. This technique has been widely used to load RNA therapeutics into exosomes without compromising their integrity or function. Zha et al. encapsulated a plasmid encoding vascular endothelial growth factor (VEGF) into exosomes via electroporation; the gene-augmented exosomes significantly improved vascularized bone regeneration by stimulating angiogenesis in the defect site [40]. Similarly, Wang et al. demonstrated that electroporating serum-derived exosomes with ATF4 mRNA produced vesicles that, when delivered, promoted chondrocyte autophagy and protected cartilage in osteoarthritis models – an approach that could be extrapolated to bone tissue repair [125]. Sonication, on the other hand, uses ultrasonic energy to temporarily disrupt exosomal membranes, mixing the cargo into their lumen. This has proven especially effective for loading larger proteins or even enzyme complexes. For instance, researchers have loaded recombinant BMP-2 protein into exosomes by brief sonication on ice, creating BMP2-loaded vesicles that subsequently enhanced the osteogenic differentiation of preosteoblast cells. Sonication is reported to achieve higher loading efficiency than electroporation for certain hydrophilic macromolecules, though it must be used cautiously to avoid damaging the exosome membrane. Other exogenous loading techniques include chemical permeabilization and simple incubation methods [126]. Using such approaches, investigators have successfully loaded exosomes with diverse therapeutic agents – from anti-inflammatory drugs to nucleic acid aptamers – broadening the scope of exosome therapy. For example, M-Exos have been infused with osteoclast-inhibiting small molecules to confer additional anti-resorptive activity alongside their native cargo, resulting in a synergistic bone-protective effect in preclinical models.

Endogenous and exogenous cargo-loading should be viewed as complementary engineering “dials” rather than competing solutions: the former reprograms the producer macrophage to bias sorting during vesicle biogenesis, preserving co-evolved packaging features that can potentiate signaling in recipient cells, whereas the latter treats purified M-Exos as a modular delivery chassis that can be functionalized post-production with diverse nucleic acids, proteins, or small molecules. Endogenous approaches tend to yield more physiologically integrated payloads but may introduce donor-cell–dependent variability and more complex CMC control; exogenous methods offer rapid adaptability and product-line flexibility, but require stringent management of membrane perturbation, cargo heterogeneity, and residual free drug/nucleic acid. Moving toward clinical-grade M-Exos will therefore demand mechanism-anchored potency assays, quantitative structure–activity relationships, and scalable GMP workflows with orthogonal release criteria that capture identity, purity, and functional activity. With these benchmarks in place, engineered M-Exos could mature from compelling biological observations into reproducible, regulatable therapeutics for bone regeneration, with representative examples summarized in Table 2.

**Table 2:** Engineered M-Exos in bone regeneration

Exosomal Cargo	Cargo Class	Macrophage Engineering Strategy	Target Bone Disease Model	Mechanistic Highlights	Ref.
miR-486-5p	microRNA	IL-4 M2 polarization of macrophages	Osteoporosis (OVX mouse model)	Exosomal miR-486-5p transferred to BMMSCs targets SMAD2, tipping the TGF-β/Smad2 pathway toward osteogenesis (while inhibiting adipogenesis), thereby rescuing bone mass in osteoporotic mice.	[102]
miR-93-5p	microRNA	IL-4 M2 polarization (miR-93-5p enriched; further lentiviral overexpression)	Osteonecrosis of the femoral head (ischemic bone necrosis)	Exosomal miR-93-5p was identified as the chief effector in M2 exos for ONFH. miR-93-5p delivered to the injury site reduces neutrophil extracellular trap (NET) formation and enhances angiogenesis in endothelial cells, fostering revascularization of the necrotic bone.	[4]
miR-365–2-5p	microRNA	IL-4 M2 polarization (naturally enriched in M2 exos; donor cells transfected to overexpress or knockdown miR-365–2-5p)	Bone defect healing (critical-sized bone injury model)	Exosomal miR-365–2-5p promotes osteoblast differentiation and bone formation. It directly binds the 3′UTR of *OLFML1* (olfactomedin-like 1), a negative regulator of osteogenesis, thereby upregulating Runx2/BMP2 and enhancing osteogenic marker expression in osteoblasts.	[94]
miR-690	microRNA	IL-4 M2 polarization (donor treated with miR-690 inhibitor to validate role)	Osteoporosis (bone loss model, in vitro BMSC differentiation assays)	Exosomal miR-690 from M2 macrophages skews MSC fate toward osteoblasts over adipocytes. In target BMSCs, miR-690 upregulates IRS-1 and TAZ (WWTR1), activating pro-osteogenic signaling. Knocking down miR-690 in donor cells blunts the exosomes’ effect, confirming that the miR-690/IRS-1/TAZ axis drives increased osteogenesis and decreased adipogenesis.	[127]
miR-1227-5p	microRNA	IL-4 M2 polarization of macrophages	Periodontitis (excessive osteoclast activity in vitro and in vivo)	Exosomal miR-1227-5p is transferred to osteoclast precursors and markedly suppresses RANKL-induced osteoclastogenesis. miR-1227-5p directly targets the osteoclast-associated receptor OSCAR, downregulating this pro-osteoclast factor. Inhibition of miR-1227-5p abolishes the exosomal effect, confirming that M2-Exos mitigate inflammatory bone loss by delivering miR-1227-5p to inhibit osteoclast differentiation.	[99]
miR-21-5p	microRNA	IFN-γ + LPS (M1) polarization + miR-21a-5p mimic transfection	Bone defect (rat calvarial defect model)	Targets GATA2 in osteoblasts to promote their differentiation, accelerating new bone formation	[68]
miR-26a-5p	microRNA	IL-4 (M2) polarization (endogenous enrichment)	Bone fracture healing (BMSC osteogenic differentiation, in vitro)	Enhances osteogenic differentiation of BMSCs (upregulating ALP, RUNX2, OPN) and suppresses adipogenesis	[38]
miR-26b-5p	microRNA	M2 polarization (IL-4-induced M2 macrophages secrete exosomes enriched in miR-26b-5p)	Osteoarthritis (cartilage protection)	M2-derived exosomal miR-26b-5p has dual targets: it inactivates TLR3 signaling in macrophages to curb M1 polarization, and also suppresses COL10A1 in chondrocytes to reduce hypertrophic degeneration. Through this miR-26b-5p/TLR3 & COL10A1 axis, M2-Exos reprogram the joint microenvironment, diminishing inflammation and chondrocyte hypertrophy, ultimately ameliorating osteoarthritic cartilage damage.	[66]
miR-155	microRNA	M1 polarization (classically activated M1 macrophages naturally secrete miR-155–enriched exosomes)	Inflammatory bone loss (osteolysis/osteoporosis)	Exosomal miR-155 from M1 macrophages impairs bone formation by downregulating osteogenic signaling molecules. It directly inhibits the expression of key pro-osteogenic factors BMP2, BMP9, and RUNX2 in osteoprogenitor cells. This leads to attenuated osteoblast differentiation and, in chronic inflammation or estrogen deficiency, contributes to net bone loss (by tipping the balance toward resorption).	[128]
miR-98	microRNA	M1 polarization (LPS/IFN-γ activation yields M1-Exos with high miR-98)	Postmenopausal osteoporosis	M1 macrophage–derived exosomal miR-98 downregulates DUSP1, a negative regulator of JNK signaling. The resulting unchecked JNK activation suppresses osteoblast differentiation (via reduced ALP, Runx2) and exacerbates osteoclastic bone resorption, thereby aggravating bone loss.	[129]
miR-221-3p	microRNA	Transfection (tumor-associated macrophages transfected with miR-221-3p mimic or inhibitor to load exosomes)	Osteosarcoma (growth & metastasis)	M2-polarized TAM exosomal miR-221-3p is delivered to osteosarcoma cells and suppresses SOCS3, leading to upregulation of JAK2/STAT3 signaling. The activated STAT3 pathway promotes tumor cell proliferation, invasion, and metastasis; conversely, inhibiting exosomal miR-221-3p or restoring SOCS3 mitigates osteosarcoma progression.	[130]
let-7a	microRNA	TAM isolation (exosomes from tumor-associated M2 macrophages naturally enriched in let-7a)	Osteosarcoma (lung metastasis)	Exosomal let-7a from TAMs is transferred to osteosarcoma cells and downregulates C15orf41, a factor that normally restrains motility. Let-7a-enriched exosomes thereby enhance osteosarcoma cell migration and invasion, worsening metastasis; overexpression of C15orf41 in tumor cells can reverse these pro-metastatic effects.	[36]
miR-378a	miRNA	Intrinsic cargo of M2-Exos – identified via sequencing; tested via miR-378a mimic on MSCs	Cranial bone defect (rat)	Exosomal miR-378a, enriched in M2-like macrophage EVs, activates the BMP2/Smad5 osteogenic signaling pathway. Administration of a miR-378a mimic (at levels reflecting M2-Exos) increased expression of BMP2, RUNX2 and other osteogenic genes in MSCs, promoting significantly greater bone volume regeneration in skull defects. (This miRNA partly underlies M2-Exos’ bone-healing effects.)	[41]
antagomir-188	siRNA (anti-miR oligonucleotide)	Exosome–liposome hybrid: Exosomes displaying CXCR4 were fused with liposomes carrying antagomir-188	Age-related osteoporosis (bone loss in aged mice)	Engineered exosomes targeted to bone marrow via CXCR4 delivered antagomiR-188 to BMSCs. This silenced miR-188 in MSCs, thereby shifting differentiation from adipogenesis to osteogenesis. Treatment reversed age-related trabecular bone loss and reduced cortical porosity in osteopenic mice.	[115]
anti-miR-6359 (EXOmotif-tagged)	anti-miR (inhibitory RNA)	EXOmotif nucleic acid engineering: anti-miR-6359 sequence modified with a 3′ EXOmotif (CGGGAGC) to enrich its packaging into exosomes	Glucocorticoid-induced osteoporosis (valproic acid-induced bone loss model in mice)	Engineered small EVs carrying anti-miR-6359 selectively target osteoclast precursors and deliver the inhibitor. This suppresses osteoclast formation and activity (via restoring SIRT3 and reducing ROS/MAPK signaling), preventing bone loss caused by valproate. Treatment protected bone mass in VPA-treated mice (but not in OVX or steroid models, indicating specificity).	[131]
LIFR-AS1	lncRNA	Transfection (TAMs transfected with shRNA/siRNA to knock down exosomal LIFR-AS1)	Osteosarcoma (tumor growth & invasion)	Exosomal lncRNA LIFR-AS1 from M2 TAMs acts as a competing endogenous RNA, sponging tumor-suppressor miR-29a in osteosarcoma cells. This releases NFIA (miR-29a target) from inhibition, leading to increased NFIA expression. The result is enhanced osteosarcoma cell proliferation and invasion, as silencing LIFR-AS1 in exosomes or restoring miR-29a markedly suppresses tumor progression.	[132]
LOC103691165	lncRNA	Polarization & overexpression (macrophages polarized to M1 or M2; exosomal lncRNA LOC103691165 further validated by overexpression/knockdown)	Bone fracture repair (rat tibial fracture model)	Exosomal lncRNA LOC103691165 (novel long non-coding RNA) is highly expressed in both M1-Exos and M2-Exos during early fracture repair. This lncRNA promotes osteogenic differentiation of BMSCs in the fracture microenvironment (likely by sponging an unknown miRNA), as both pro-inflammatory and anti-inflammatory macrophages secrete it to enhance callus formation and bone regeneration in a rat fracture model.	[91]
IL-10 mRNA	mRNA (coding for IL-10 cytokine)	IL-4 M2 polarization (“reparative” macrophages)	Periodontitis (murine model of alveolar bone loss)	Exosomes deliver IL-10 mRNA into target cells (BMSCs and osteoclast precursors), raising IL-10 levels and activating IL-10/IL-10R signaling. This shifts differentiation: promoting osteoblastogenesis and suppressing osteoclastogenesis, thereby protecting against inflammatory bone resorption.	[39]
Melatonin (Mel@M2-Exos)	Small molecule (hormone)	M2-Exos loaded with melatonin (preconditioned + ultrasonic encapsulation)	Periodontitis (rat ligature-induced model)	Melatonin-loaded exosomes locally release melatonin, which alleviates ER stress and drives an M1→M2 immune switch in periodontal lesions. This reduces inflammatory damage and restores the osteogenic/cementogenic capacity of periodontal ligament cells, promoting regeneration of alveolar bone.	[133]
Glutamate	Small-molecule metabolite (amino acid)	IL-4 M2 polarization (M2-EVs naturally enriched in glutamate)	Osteoporosis (OVX-induced bone loss in mice)	M2 macrophage-derived EVs deliver glutamate to osteoclast precursors (OCPs). The glutamate uptake in OCPs boosts glutamine metabolism and production of α-ketoglutarate, which triggers Jmjd3-dependent epigenetic reprogramming. This process converts OCPs into M2-like macrophage cells instead of osteoclasts, thereby rebalancing bone remodeling and alleviating osteoporosis.	[134]
Hydrogen sulfide (H_2_S)	Protein	IL-4 M2 polarization + H2S donor (GYY4137) preconditioning of macrophages	Bone regeneration (critical-size calvarial defect in mice)	H2S stimulation of macrophages (via GYY4137) increases exosomal Moesin content. This cytoskeletal-linker protein on M2-Exos facilitates their endocytosis by MSCs in the bone defect, thereby amplifying exosome uptake. The moesin-rich exosomes potently activate β-catenin signaling in MSCs, driving osteogenic differentiation and markedly improving bone defect repair.	[123]
CYLD protein	Protein (deubiquitinase)	IL-4 M2 polarization of macrophages	Inflammatory osteolysis (e.g. periodontitis or osteolytic lesions)	M2-derived exosomes deliver the CYLD deubiquitinase into osteoclast precursor cells. CYLD in the recipient osteoclasts inactivates STAT3 signaling, a pathway necessary for osteoclast formation and function. By shutting down STAT3, exosomal CYLD halts osteoclast differentiation and bone resorption, thereby protecting bone structure in inflammatory conditions.	[135]
BMSC-specific aptamer	RNA aptamer (targeting ligand)	Exogenous surface functionalization (3WJ RNA nanoparticle conjugating a bone-marrow MSC–targeting aptamer to exosome surface)	Bone fracture (mouse femoral fracture model)	Confers targeting ability to M2-Exos – functionalized exosomes home to bone injury sites and gradually release their natural cargo – leading to higher accumulation at the fracture and enhanced bone regeneration compared to unmodified exosomes	[111]
Linezolid	Small-molecule drug (antibiotic)	Exogenous loading (incubation of M-Exos with drug at 37 °C for passive encapsulation)	Bone infection (intracellular MRSA infection model)	Delivers antibiotic into infected cells to eradicate intracellular *S. aureus* that evade free drugs, achieving superior infection clearance in vitro and in vivo compared to free linezolid	[136]
CXCL12 induction (no distinct cargo)	M2-Exos inducing host factor	IL-4-induced M2-Exos (unmodified) applied to stem cells under inflammatory conditions (LPS)	Periodontal bone regeneration (in vitro hPDLSCs; periodontitis model)	M2-Exos upregulate CXCL12 (SDF-1) expression in LPS-challenged periodontal ligament stem cells. Elevated SDF-1 restored the cells’ osteogenic potential and reduced inflammatory cytokines. As a result, M2-Exos treatment drove osteogenic differentiation of hPDLSCs (increased ALP, Runx2, OCN) and lowered NF-κB-mediated inflammation. Knockdown of CXCL12 abrogated these effects, confirming exosomal induction of SDF-1 as the mechanism. This suggests M2-Exos could promote periodontal bone regeneration by recruiting/protecting osteoprogenitors via SDF-1.	[137]
M2-Exos (unenriched)	mixed cargo	M2 phenotype induction (collect exosomes from IL-4-polarized M2 macrophages; used directly)	Diabetic fracture repair (STZ-induced diabetic rat)	M2-Exos act as natural immunomodulators in the diabetic injury milieu. They decrease pro-inflammatory M1 macrophage numbers and cytokines at the fracture site, skewing the osteoimmune environment toward healing. This improved immune balance accelerates callus formation and mineralization in diabetic rodents, overcoming the chronic inflammation that impairs fracture healing.	[74]
M2-Exos (mixed cargo)	mixed cargo	M2 phenotype induction (exosomes from M2 macrophages integrated into scaffold)	Osteonecrosis of femoral head (ONFH)	M2-Exos have been applied to ischemic bone heads to modulate local inflammation and stimulate repair. These exosomes reduce neutrophil-driven tissue damage and encourage reparative angiogenesis by communicating with endothelial cells. In an ONFH model, systemically delivered M2-Exos homed to the femoral head and improved revascularization and new bone formation, highlighting their therapeutic efficacy in regenerating necrotic bone.	[138]
“RAB” Peptide (ROS-activated bone-targeting antimicrobial peptide)	Peptide	Click chemistry conjugation of a tri-block peptide (bone-targeting moiety + ROS-cleavable linker + antimicrobial peptide) onto exosome surface (termed RAB-EXO)	Osteomyelitis (bacterial bone infection in rats)	RAB-EXOs target infected bone tissue and, in the high-ROS osteomyelitic microenvironment, cleave to release antimicrobial peptides, killing bacteria (MRSA/E. coli) and resolving infection. Exosomes simultaneously polarize macrophages to M2, reducing inflammation and promoting bone healing; complete infection clearance and bone defect repair achieved in 28 days.	[139]
M2-Exos in dual-sensitive hydrogel	native exosome cocktail	Responsive hydrogel encapsulation: M2-Exos were embedded in an adhesive hydrogel that is pH/ROS dual-sensitive for sustained, on-demand release in the injury site	Large bone defect repair (rat cranial defect)	The hydrogel-protected exosomes maintained bioactivity and were gradually released in response to inflammatory pH/ROS at the defect. This promoted a pro-regenerative microenvironment: sustained exosome release enhanced osteogenesis and angiogenesis while dampening inflammation, yielding significantly improved bone defect repair (greater mineralized bone volume and vessel formation) compared to free exosomes.	[140]
M2-Exos (via scaffold)	mixed cargo	Electrospun biomimetic periosteum + exosomes: M2-Exos were incorporated into an electrospun polycaprolactone/gelatin membrane (simulating periosteum)	Critical-sized bone defect (segmental defect in rabbits)	The exosome-loaded periosteum scaffold provided a combinatorial therapy: it recruited host stem cells and immune cells to the defect, enhanced M2 polarization and angiogenesis, and provided osteoinductive signals. This resulted in robust bone regeneration bridging large defects, outperforming scaffolds or exosomes alone. The engineered periosteum promoted orderly regeneration with mature vasculature and new bone tissue formation.	[87]

### Biomaterials scaffold-mediated controlled and sustained release

**Fig. 4:**
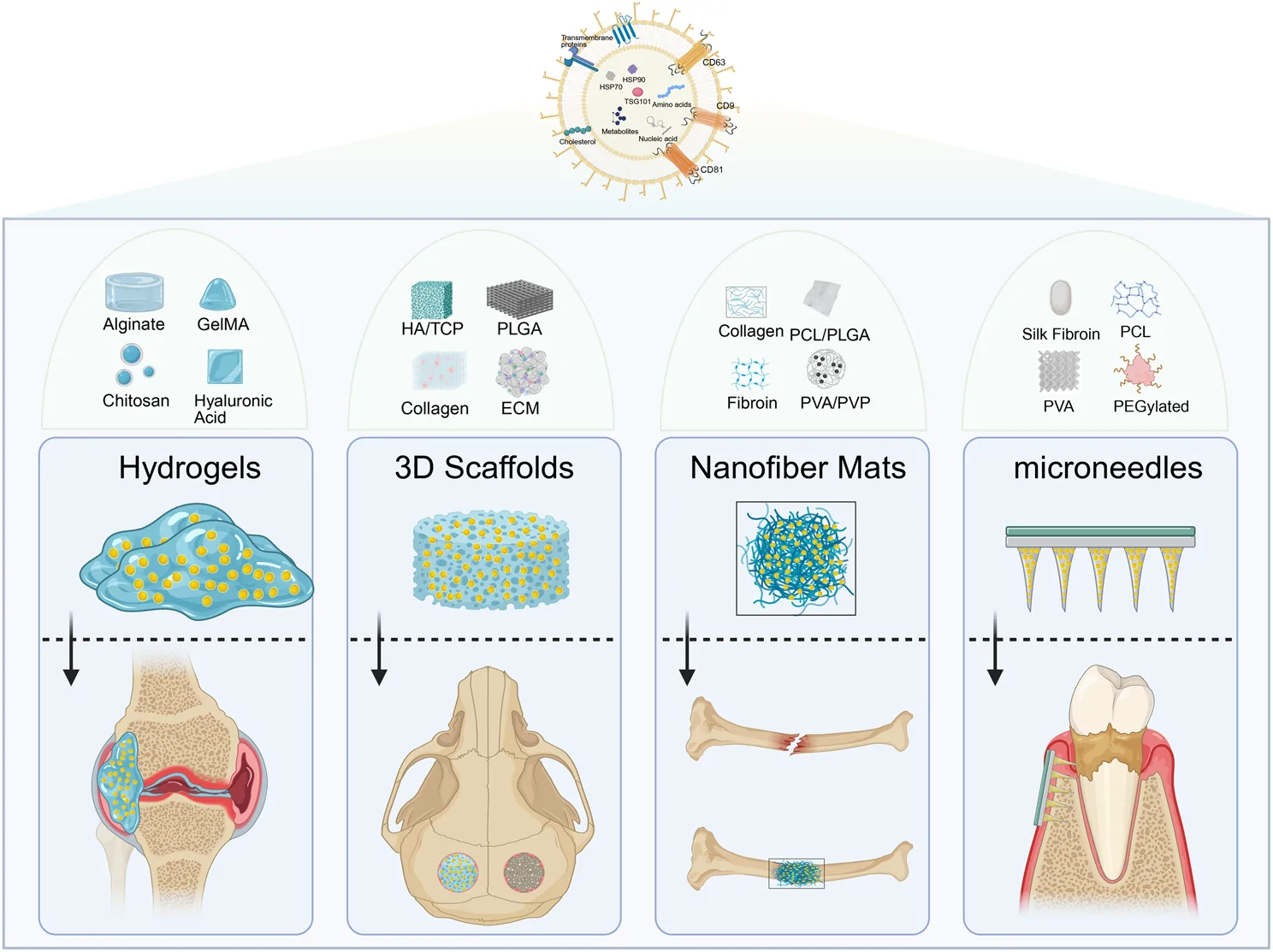
Biomaterial-integrated platforms for sustained, localized exosome release in bone regeneration. Illustrative depiction of scaffold- and hydrogel-based systems engineered for the localized delivery and retention of M-Exos at bone defect sites. These platforms encapsulate M-Exos to shield against enzymatic degradation and enable controlled, prolonged release of therapeutic cargo, fostering a concentrated regenerative niche. Exemplary designs encompass porous osteoconductive scaffolds, nanoscale surface coatings, and stimuli-responsive hydrogels that trigger exosome discharge in response to microenvironmental cues (e.g., pH, ROS). Such localized strategies augment cellular uptake, prolong immunomodulatory and pro-osteogenic effects, and minimize systemic off-target exposure, thereby enhancing therapeutic outcomes in bone defect repair

A persistent challenge with any soluble factor therapy—including exosomes—is ensuring that these biologic cues remain at the injury site long enough to exert their regenerative effects. Simply injecting exosomes into a fracture or into the bloodstream often leads to rapid dispersion or clearance, which markedly lowers the effective dose at the target tissue. To address this challenge, researchers have begun integrating exosomes into various biomaterial scaffolds, creating composite grafts that provide both structural support and localized delivery of exosomes, as illustrated in Fig. 4. This concept of an “exosome-functionalized scaffold” essentially combines the benefits of classical tissue engineering with the potent paracrine signaling of cell-free biologics [141].

One illustrative strategy is a periosteum-mimetic electrospun nanofiber scaffold loaded with M2-Exos. The periosteum—the membrane covering bone—is rich in osteoprogenitor cells and healing factors. By mimicking the periosteum with a fibrous scaffold enriched with anti-inflammatory M2-Exos, the aim is to recreate that osteogenic and angiogenic microenvironment at a defect site. In a preclinical study, wrapping a critical-sized bone defect with this exosome-functionalized periosteal scaffold significantly improved bone healing compared to an identical scaffold without exosomes. The released vesicles recruit endogenous repair cells, modulate the local immune response, and directly stimulate angiogenesis and osteogenesis within the defect [87]. The combination of a physical scaffold with exosomes thus addresses two critical requirements simultaneously: the scaffold provides mechanical stability and a 3D template for new tissue growth, while the exosomes supply instructive biochemical cues that guide cell recruitment and differentiation.

Beyond fibrous scaffolds, hydrogels have also been widely explored as exosome carriers. Injectable hydrogels—particularly thermosensitive ones that solidify in situ—can encapsulate exosomes and gradually release them at the target site. For example, in a periodontal bone defect model, a thermosensitive chitosan-based hydrogel loaded with M-Exos conformed to the irregular defect and retained the vesicles locally, leading to significantly better bone regeneration than delivering exosomes in solution alone [142]. Similarly, other biomaterials such as mineralized bone cements and metal–organic frameworks (MOFs) have been impregnated with exosomes to create “EV-functionalized” bone fillers [143]. In all these cases, the biomaterial scaffold protects the exosomes from rapid clearance and localizes them at the defect, thereby increasing the local concentration and prolonging the duration of exosome activity.

Recent studies have begun to demonstrate this translational potential. In a rat alveolar bone defect model, Lou et al. reported improved regenerative outcomes following exosome-based scaffold treatment compared with scaffold treatment alone [144]. Similarly, delivering hiPSC-MSC-derived exosomes via a β-tricalcium phosphate (β-TCP) scaffold markedly enhanced the healing of critical-size calvarial defects in an osteoporotic rat model [138]. These findings reinforce the notion that exosome-functionalized materials can transform otherwise inert scaffolds into bioactive implants capable of orchestrating robust bone tissue repair [145].

Collectively, integrating exosomes into biomaterial scaffolds has been shown to markedly amplify bone regeneration outcomes compared to scaffolds or exosomes alone. By spatially immobilizing exosomal cues at the repair site, composite graft systems can establish a localized pro-regenerative microenvironment that supports coordinated bone healing responses. Importantly, owing to their superior physicochemical stability relative to many recombinant growth factors, exosomes can be readily stored or lyophilized with scaffold materials while largely preserving bioactivity, underscoring their potential for the development of off-the-shelf therapeutic implants. Key challenges such as scalable manufacturing and long-term exosome stability in implants still need to be addressed, but the robust preclinical results demonstrate significant translational promise. This emerging strategy paves the way for “smart” bone grafts pre-loaded with exosomes – ready-to-use, cell-free implants capable of directing and accelerating bone repair in patients.

## Clinical translation challenges and future outlook for M-Exos

### Manufacturing standardization and quality control

The clinical translation of M-Exos is fundamentally limited by the absence of manufacturing and quality control frameworks that are compatible with regulatory evaluation. While their biological activity has been extensively demonstrated at the preclinical level, clinical development requires a shift from descriptive characterization toward product-oriented definition. Adopting Good Manufacturing Practice (GMP) principles provides a roadmap to clinical-grade production, enforcing reproducibility, traceability, and quality through controlled processes and detailed documentation. Under GMP, every step – from cell culture to purification – is conducted in certified facilities by trained personnel with comprehensive batch records, ensuring the process is auditable and meets regulatory expectations [146]. However, physicochemical and molecular characterization alone is insufficient to ensure consistent therapeutic activity. Accordingly, mechanism-informed functional (potency) assays are required to capture the intended biological effects of M-Exos—such as immunomodulatory capacity, osteoinductive activity or pro-angiogenic support—and to translate these activities into actionable batch-release specifications. These functional readouts provide a practical basis for defining batch release criteria and for ensuring lot-to-lot comparability, thereby transforming heterogeneous vesicle preparations into regulatable therapeutic products. Researchers have begun addressing these gaps by implementing quality control checkpoints – for example, one study produced clinical-grade mesenchymal exosome batches with documented lot-to-lot consistency in microRNA/protein profiles and six-month stability at −80°C [170]. These efforts underscore that rigorous standardization in exosome isolation, purification, and characterization is both possible and necessary to ensure the safety and efficacy of M-Exos therapeutics.

### Scaling up production

Achieving industrial-scale exosome production remains a significant challenge. Primary macrophages exhibit limited capacity for expansion, and their exosome yield per cell is typically low [147]. Thus, researchers are exploring cell-engineering approaches and advanced culture systems to boost production [148]. For example, 3D bioreactors and scaffold-based cultures can significantly increase exosome output per cell [149]. These high-yield systems, combined with efficient purification methods like tangential flow filtration and size-exclusion chromatography [150, 151], aim to supply milligram-to-gram quantities of exosomes without sacrificing quality [152]. Ensuring consistent product quality during large-scale exosome manufacturing necessitates rigorous monitoring of critical quality attributes (CQAs) [153]. Notably, regulatory bodies are already engaging with this challenge – the Japanese PMDA’s Science Board, for instance, has issued guidance on large-scale EV manufacturing and quality requirements [154]. Ongoing refinement of scalable and GMP-compliant manufacturing platforms will be crucial to translating M-Exos research into viable, clinical-grade therapeutic products [155].

### In vivo delivery efficiency and safety considerations

Even with high-quality exosome preparations, effective in vivo delivery to target tissues like bone remains a key challenge [156]. Systemically administered exosomes often undergo rapid clearance by the mononuclear phagocyte system, including the liver and spleen, limiting the fraction reaching the intended site [157]. Autologous exosomes may have a longer circulation time by evading immune recognition and uptake. However, simply relying on passive homing is usually insufficient for precise delivery to injury sites or specific cell populations [158]. To address this, researchers are developing targeted delivery strategies. Intriguingly, exosomes inherently carry surface adhesion proteins and integrins that can confer some tissue tropism [159]. For instance, exosomes of melanoma origin have been shown to preferentially accumulate in lymph nodes, reflecting their inherent organotropic properties [160]. Building on this concept, engineered exosomes with enhanced bone-targeting capability have emerged [161]. Surface modification of exosomes with bone-homing ligands or membrane fusion proteins can improve their accumulation in skeletal tissues [162]. Recent advances have shown that such modified exosomes exhibit strong bone-targeting and can deliver cargos more efficiently to bone lesions, thereby improving therapeutic efficacy while reducing off-target effects [163]. Local delivery approaches are also being pursued: for example, incorporating exosomes into biomaterial scaffolds or hydrogels that are implanted at the injury site provides a sustained, localized release. This strategy can bypass systemic barriers and has demonstrated improved bone regeneration outcomes in preclinical models [164]. Taken together, enhancing delivery – via either systemic modifications or local biomaterial-assisted administration – is critical to maximizing the therapeutic index of M-Exos.

### Safety, immunocompatibility, and long-term risks

By virtue of their natural origin, exosomes are biocompatible and exhibit low intrinsic immunogenicity [165]. M-Exos carry membrane proteins including MHC molecules and costimulatory factors that could stimulate an immune response if donor and host are mismatched [166]. Therefore, careful donor selection, or even gene-editing to remove immunogenic epitopes, may be needed for off-the-shelf exosome products [167]. In preclinical toxicology studies, repeated administration of high-dose exosomes has so far shown minimal adverse effects [168]. For example, a month-long dosing of MSC-derived EVs in rats caused no significant toxicity or organ damage [169]. Early clinical trials have also hinted at a favorable safety profile: in a Phase I study of dendritic cell-derived exosomes in cancer patients, no dose-limiting toxicities were observed, suggesting that exosome administration was well-tolerated [170]. These findings support the notion that exosome therapies can be safe, but comprehensive safety assessment remains imperative. Long-term risks, such as unforeseen immunological reactions or pro-tumorigenic effects, need careful monitoring. Exosomes carry diverse bioactive cargos, and while this underlies their therapeutic potency, it also means there is a complex mechanism of action that must be understood to avoid off-target or systemic side effects [44]. Thus, rigorous safety pharmacology and immunogenicity testing in multiple animal models – and eventually in human trials – is required to fully de-risk M-Exos therapies for clinical use.

### Regulatory and translational landscape

**Fig. 5:**
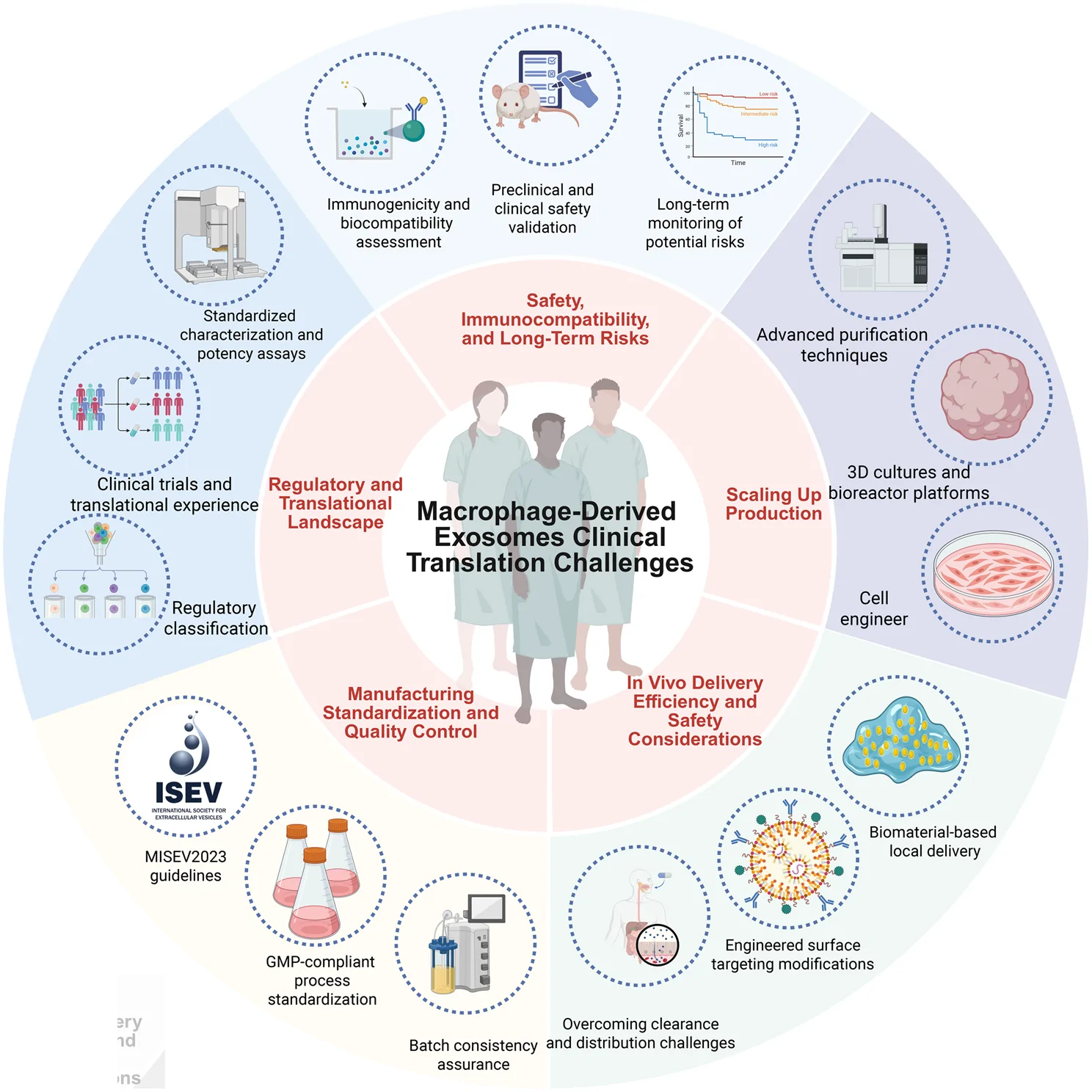
Translational challenges and solution-oriented considerations for M-Exos therapies

As the field progresses, regulatory agencies have started grappling with how to classify and evaluate exosome-based therapeutics. In the United States, exosome products intended to treat human disease or condition are regulated as drugs and biological products there are currently no FDA-approved [171]. This means that clinical development generally requires an Investigational New Drug (IND) pathway and formal demonstration of safety, quality, and efficacy. In Europe, the regulatory route is product-specific; depending on composition, source, degree of manipulation, and mechanism of action, an advanced-therapy or biologics-related framework may become relevant [172]. Accordingly, the path to clinical translation will require not only stronger efficacy data but also clearer product definitions, standardized manufacturing controls, validated potency assays, and early regulatory engagement. Encouragingly, a growing number of exosome-based therapeutics are entering formal development pipelines. Dozens of clinical trials worldwide are now investigating EV therapies in indications ranging from cancer and neurodegenerative disorders to tissue repair. The lessons learned from these initial trials will directly shape future regulatory expectations, ultimately laying the foundation for standardized guidelines and best practices that will accelerate the safe and effective translation of exosome therapeutics, as summarized in Fig. 5.

This schematic summarizes the major barriers and corresponding solution pathways for the clinical translation of M-Exos as bone regenerative therapeutics. Key manufacturing challenges include scalable, GMP-compliant production, batch-to-batch consistency, and robust quality control, which can be addressed through standardized isolation workflows, validated potency assays, and harmonized cargo characterization metrics. Safety and biocompatibility considerations encompass immunogenicity, biodistribution, and long-term risk monitoring, underscoring the need for rigorous preclinical evaluation and longitudinal safety assessment. Translational implementation further requires optimization of in vivo delivery efficiency, dosing strategies, and local retention, supported by biomaterial-assisted delivery systems and engineered surface modifications. Advances in upstream cell engineering, 3D culture systems, and bioreactor-based expansion provide scalable solutions for production, whereas alignment with evolving regulatory frameworks and international guidelines (such as MISEV) is essential for regulatory approval. Collectively, integrating standardized manufacturing, safety validation, delivery optimization, and regulatory harmonization offers a practical roadmap for advancing M-Exos toward clinically viable, cell-free bone regeneration therapies.

## Conclusion

This review systematically elucidates the regulatory roles of M-Exos across the four established stages of bone healing – the inflammatory phase, soft callus formation, hard callus formation, and remodeling. By organizing current literature along this timeline and integrating breakthroughs from recent macrophage single-cell RNA-sequencing datasets, this work establishes a rigorous spatiotemporal framework demonstrating how M-Exos coordinate and fine-tune the immunomodulatory microenvironment at each repair stage. This unique stage-by-stage analysis highlights that M-Exos are not mere bystanders but active modulators throughout bone regeneration, coordinating inflammation resolution, promoting tissue formation, and ultimately contributing to effective remodeling of bone. Such an approach provides a comprehensive and dynamic understanding of their multifaceted contributions, laying a conceptual foundation for timing-specific therapeutic strategies that leverage exosomal signals in bone repair.

Viewed through this lens, M-Exos constitute a compelling therapeutic paradigm that integrates endogenous intercellular messaging with the tunability of engineered biologics. By strategically modulating the bone healing cascade in both space and time, these nanoscale vesicles hold the promise of accelerating fracture repair and improving healing outcomes in ways conventional therapies cannot. Although challenges persist, the continued refinement of exosome engineering techniques and a clearer roadmap for clinical implementation are bringing us closer to translating this spatiotemporal exosome strategy from bench to bedside. The advances made and highlighted in this review provide not only a clearer picture of the current landscape but also a forward-looking vision: with rigorous research and innovation, M-Exos therapies are poised to evolve from experimental concept to a transformative clinical reality in bone healing and regenerative medicine [173, 174].

## Electronic supplementary material

Below is the link to the electronic supplementary material.

Supplementary material 1

## Data Availability

Not applicable.

## Abbreviations

ALIX: ALG-2-interacting protein X
ALN: Alendronate
ALP: Alkaline phosphatase
BMSCs: Bone marrow mesenchymal stem cells
BMP: Bone morphogenetic protein
BMP2 (BMP-2): Bone morphogenetic protein 2
CD9: Cluster of differentiation 9
CD63: Cluster of differentiation 63
CD81: Cluster of differentiation 81
CD86: Cluster of differentiation 86
COL10A1: Collagen type X alpha 1 chain
CXCL12: C-X-C motif chemokine ligand 12
CXCR4: C-X-C chemokine receptor type 4
CYLD: CYLD lysine 63 deubiquitinase
DSS: Asp–Ser–Ser (bone-targeting peptide motif)
ECM: Extracellular matrix
EMA: European Medicines Agency
EV(s): Extracellular vesicle(s)
IFN-γ: Interferon gamma
IL-1β: Interleukin-1 beta
IL-4: Interleukin-4
IL-6: Interleukin-6
IL-10: Interleukin-10
M-Exos: Macrophage-derived exosomes
M1-Exos: M1 macrophage-derived exosomes
M2-Exos: M2 macrophage-derived exosomes
MHC: Major histocompatibility complex
miRNA: microRNA
mRNA: messenger RNA
NF-κB: Nuclear factor kappa B
OCN: Osteocalcin
RUNX2: Runt-related transcription factor 2
SDF-1: Stromal cell-derived factor 1
siRNA: Tmall interfering RNA
TEM: Transmission electron microscopy
TGF-β1: Transforming growth factor beta 1
TLR4: Toll-like receptor 4
TNF-α: Tumor necrosis factor alpha
TSG101: Tumor susceptibility gene 101
VEGF: Vascular endothelial growth factor
